# Mechanochemical Synthesis of New Praziquantel Cocrystals: Solid-State Characterization and Solubility

**DOI:** 10.1021/acs.cgd.4c00296

**Published:** 2024-05-11

**Authors:** Marieta Mureşan-Pop, Simion Simon, Ede Bodoki, Viorica Simon, Alexandru Turza, Milica Todea, Adriana Vulpoi, Klara Magyari, Bogdan C. Iacob, Alexandra Iulia Bărăian, Mateusz Gołdyn, Clara S. B. Gomes, Margarida Susana, M. Teresa Duarte, Vânia André

**Affiliations:** † Nanostructured Materials and Bio-Nano Interfaces Department, Interdisciplinary Research Institute on Bio-Nano-Sciences, Babes-Bolyai University, 42, Treboniu Laurian, Cluj-Napoca 400271, Romania; ‡ INSPIRE Research Platform, Babes-Bolyai University, 11, Arany Janos, Cluj-Napoca 400028, Romania; § Analytical Chemistry Department, Faculty of Pharmacy, Iuliu Haţieganu University of Medicine and Pharmacy, 4, Louis Pasteur, Cluj-Napoca 400349, Romania; ∥ Mass Spectrometry, Chromatography and Applied Physics Department, National Institute for Research and Development of Isotopic and Molecular Technologies, Cluj-Napoca 400293, Romania; ⊥ Molecular Sciences Department, Faculty of Medicine, Iuliu Haţieganu University of Medicine and Pharmacy, 4, Louis Pasteur, Cluj-Napoca 400349, Romania; # Faculty of Chemistry, Adam Mickiewicz University in Poznań, Uniwersytetu Poznańskiego 8, Poznań 61-614, Poland; ∇ Center for Advanced Technology, Adam Mickiewicz University in Poznań, Uniwersytetu Poznańskiego 10, Poznań 61-614, Poland; ○ LAQV-REQUIMTE, Department of Chemistry, NOVA School of Science and Technology (NOVA FCT), NOVA University of Lisbon, Caparica 2829-516, Portugal; ◆ Centro de Química Estrutural, Institute of Molecular Sciences, Instituto Superior Técnico, Universidade de Lisboa, Av. Rovisco Pais, Lisboa 1049-001, Portugal; ¶ Associação do Instituto Superior Técnico para a Investigação e Desenvolvimento (IST-ID), Avenida António José de Almeida, 12, Lisboa 1000-043, Portugal

## Abstract

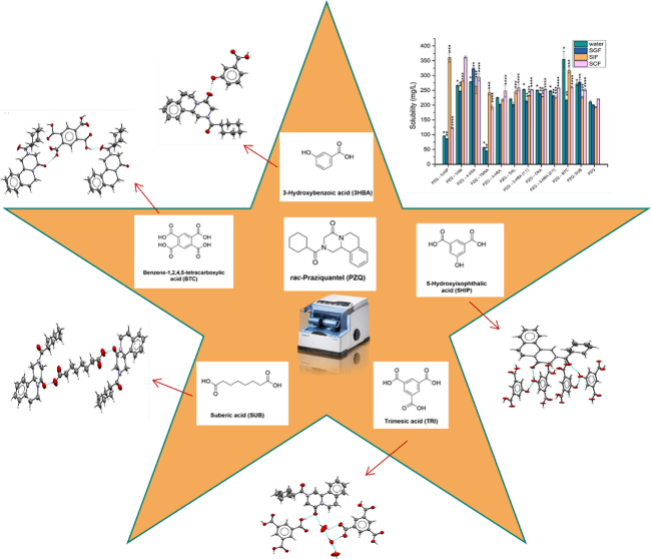

New cocrystals of praziquantel with suberic, 3-hydroxybenzoic, benzene-1,2,4,5-tetracarboxylic, trimesic, and 5-hydroxyisophthalic acids were obtained through ball milling experiments. The optimal conditions for the milling process were chosen by changing the solvent volume and the mechanical action time. Supramolecular interactions in the new cocrystals are detailed based on single-crystal X-ray diffraction analysis, confirming the expected formation of hydrogen bonds between the praziquantel carbonyl group and the carboxyl (or hydroxyl) moieties of the coformers. Different structural characterization techniques were performed for all samples, but the praziquantel:suberic acid cocrystal includes a wider range of investigations such as thermal analysis, infrared and X-ray photoelectron spectroscopies, and SEM microscopy. The stability for up to five months was established by keeping it under extreme conditions of temperature and humidity. Solubility studies were carried out for all the new forms disclosed herein and compared with the promising cocrystals previously reported with salicylic, 4-aminosalicylic, vanillic, and oxalic acids. HPLC analyses revealed a higher solubility for most of the new cocrystal forms, as compared to pure praziquantel.

## Abstract 2

Five new crystal structures of praziquantel with carboxylic acids were determined by single-crystal X-ray diffraction: three are cocrystals with 3-hydroxybenzoic acid (**PZQ·3HBA** 1:1), benzene-1,2,4,5-tetracarboxylic acid (**PZQ·BTC** 2:1), and suberic acid (**PZQ·SUB** 2:1), and two are cocrystal solvates with trimesic (**PZQ·TRI·H_2_O** 1:2:2) and 5-hydroxyisophthalic acids (**PZQ·5HIP·MeCN** 1:4:2). Praziquantel molecules show different conformations and crystal packing in cocrystals and solvate structures. All cocrystals revealed a significant solubility improvement compared to praziquantel. An increase in solubility in SIF was obtained by cocrystallization of praziquantel with all acids: by 1.87-fold at **PZQ·5HIP·MeCN**, by 1.65-fold at **PZQ·BTC**, by 1.2-fold at **PZQ·3HBA**, by 1.26-fold at **PZQ·TRI·H_2_O**, and by 1.18-fold at **PZQ·SUB**. Due to its improved solubility in the different segments of the gastrointestinal tract, **PZQ·BTC** appears to be the most promising candidate for higher absorption and increased oral bioavailability. Additionally, the nature of the **PZQ·SUB** cocrystal was confirmed by combining X-ray diffraction (powder and single-crystal) with spectroscopy techniques (FT-IR and XPS) and thermal analyses (DSC and TG). This cocrystal is stable for at least five months on storage at 40°C/75% RH. The melting points of **PZQ·SUB** are in line with its solubility, while the enthalpy decreased in the cocrystal. Our study expands the understanding of mechanochemistry while presenting alternatives for the development of more effective formulations with improved solubility, suggesting promising prospects for enhanced therapeutic efficacy of the anthelmintic compound praziquantel.

## Introduction

In the pharmaceutical industry, the development of innovative drugs with improved effects is a real necessity due to the emergence of various infectious diseases resistant to usually administered drugs. Such new drug forms can be obtained by combining an active pharmaceutical ingredient (API) with other substances, resulting in new solid forms or supramolecular complexes such as salts or cocrystals.^1−3^ Mechanochemistry is a frequent technique used in the synthesis of these new materials that display improved physicochemical properties due to its simplicity, noninvasive effects on the environment, scalability, and low process costs such as time and energy.^4−10^ Diverse experiments have shown that parameters selected in the process of mechanical mortaring, such as the vibration frequency, ball size and weight, time of mechanical action, amount of solvent, or temperature, influence the resulting outcomes.^11^ The structural and chemical changes of the materials are directly influenced by the mechanical energy stored during the process: as this energy increases, the coherence energy of the particles will decrease, resulting in the appearance of defects inside the material or even its amorphization.^12^ In 2009, Friščić et al. defined the wetting parameter of ingredients at the grinding process noted with “η” as the ratio between the volume of the solvent and the weight of the sample emphasizing the need to establish control of parameters specific to the ball milling process and which would lead to successful mechanochemical reactions.^13−15^

According to the Biopharmaceutical Classification System (BCS), the anthelmintic drug Bitricide (brand name for praziquantel, PZQ) belongs to BCS class II due to very slight solubility in water and high permeability,^16,17^ suggesting that the efficiency of PZQ in the gastrointestinal tract is directly influenced by the dissolution process. This implies the administration of a higher dose of the active substance to reach the minimum effective concentration. Additionally, fluctuating clinical efficacies were also reported based on the use of various brands of generic praziquantel formulations due to suboptimal bioequivalence.^18−22^ All these issues raised several questions, and different attempts were reported for the synthesis of new solid forms (e.g., inclusion complexes, nanoparticles, cocrystals, and solid dispersions) aiming to improve praziquantel’s solubility and its overall bioavailability upon advanced formulation strategies.^21,23−32^

**Scheme 1:**
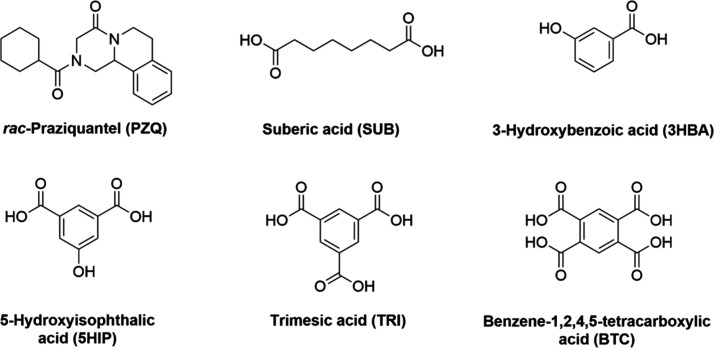
Chemical Structures of Praziquantel and the Coformers Used for Cocrystal Preparation

Based on results obtained in previous studies regarding cocrystals of praziquantel using ball milling,^24−29^ our main objective was to investigate the cocrystallization of this anthelmintic compound with different acids accepted in the GRAS (generally regarded as safe) list by the U.S. Food and Drug Administration (FDA): suberic, 3-hydroxybenzoic, 4-hydroxybenzoic, benzene-1,2,4,5-tetracarboxylic, trimesic, and 5-hydroxyisophthalic acids. The molecular structures of praziquantel and coformers used to obtain the cocrystals unveiled herein are presented in Scheme 1.

The primary goal of this work was to identify the grinding conditions leading to the cocrystallization of PZQ with the different coformers. The expectation was that during the mechanical process of liquid-assisted grinding of ingredients in the ball mill, the praziquantel would establish intermolecular hydrogen bonds with the guest molecule so that the acid molecules intercalate in the praziquantel lattice forming heterodimeric synthons.^23,33^ In the grinding process, the volume of the wetting liquid was varied as well as the process time. It is worth mentioning that, even though a cocrystal had already been reported with 3-hydroxybenzoic acid, the optimization of the conditions led to a new cocrystal with a different stoichiometry. After a deeper screening of mechanochemical conditions, also, a particularly extensive characterization was carried out for the suberic acid system, the only one enclosing a linear coformer. An important characteristic of pharmaceutical cocrystals is their ability to modify the physicochemical properties of individual molecules, and solubility is among the most targeted properties when using pharmaceutical compounds. Even though several praziquantel cocrystals had already been reported, to the best of our knowledge, there was still no broad solubility study comparing the most promising forms. Thus, the solubility of the new cocrystals disclosed herein was assessed by HPLC-UV analysis and compared with the solubility of the previously reported praziquantel cocrystals with salicylic, 4-aminosalicylic, vanillic, oxalic, 4-hydroxybenzoic, and 3-hydroxybenzoic acids.

## Experimental Section

### Materials

Praziquantel bulk powder (PZQ, MW = 312.41 g/mol) and suberic acid (SUB, MW = 174.2 g/mol) were provided by Alfa Aesar and used as received. Salicylic acid (SAL, MW = 138.122 g/mol), 3-hydroxybenzoic acid (3HBA, MW = 138.122 g/mol), 4-hydroxybenzoic acid (4HBA, MW = 138.122 g/mol), 4-aminosalicylic acid (4ASA, MW = 153.137g/mol), vanillic acid (VAN, MW = 168.148 g/mol), oxalic acid (OXA, MW = 90.034 g/mol), trimesic acid (TRI, MW = 210.140 g/mol), benzene-1,2,4,5-tetracarboxylic acid (BTC, MW = 254.150 g/mol), and 5-hydroxyisophthalic acid (5HIP, MW = 182.13 g/mol) were obtained from Sigma-Aldrich and used as received. Ethanol, dichloromethane, and buffers were obtained from VWR International and were used without any further purification. Acetonitrile (MeCN) was obtained from Sigma-Aldrich and used without further purification.

### Cocrystal Screening

A Retsch MM400-mixer mill was used for the PZQ multicomponent system preparation. Substrates with a total weight of 250 mg (256 mg for PZQ and SUB), 2 stainless-steel balls with a diameter of 4 mm, and the appropriate volume of a liquid medium were introduced into a 10 mL stainless-steel jar. Grindings were carried out for 30–60 min, at the constant frequency of 25 Hz. The volume of the liquid added to mechanical grinding was calculated from the relation of the wetting parameter η^13^ defined as the ratio between the volume of liquid (μL) and the total mass of the reactants (mg). A strict volume of acetonitrile (wetting parameter η of 0.16) was used to grind PZQ with 3HBA (2:1 and 1:1 stoichiometric ratios) and 4HBA, VAN, OXA, BTC, 4ASA, and 5HIP as coformers. PZQ·0.5H_2_O^28^ obtained by earlier PZQ grinding with water was used for water-assisted grinding with TRI (wetting parameter η of 0.32). At grinding PZQ with SUB (2:1 stoichiometric ratio), the volume of ethanol/dichloromethane (1:1 v/v) added was calculated according to the wetting parameters η of 0, 0.78, and 1.56. Details about mechanochemical synthesis are presented in Tables S1 and S2.

Before analysis, the samples were dried at 37 °C in an oven for 24 h.

### Solution Synthesis

A small amount of the white powder obtained after grinding PZQ with 3HBA in a 1:1 stoichiometric ratio was dissolved in acetonitrile at 40 °C. In the case of **PZQ·BTC** (2:1) and **PZQ·5HIP·MeCN** (1:4:2), the milling product did not dissolve completely in acetonitrile, and after 15 min of stirring, a few drops of water were added to the solution. The solution was mixed for another 15 min. A small quantity from the powder sample **PZQ·SUB** (2:1) was dissolved in a mix of solvents ethanol/dichloromethane (1:1) (v/v). After slow evaporation of the solution, single crystals of **PZQ·SUB** (2:1) suitable for X-ray single-crystal measurements were obtained. An equimolar mixture of PZQ and trimesic acid (TRI) was dissolved in an ethyl acetate-*n*-heptane mixture. This solution was heated up to 40 °C for approximately 20 min. Plate-like monocrystals of **PZQ·TRI·H**_**2**_**O** (1:2:2) were obtained on top of the solvent during the evaporation of the solution.

### Powder X-ray Diffraction (PXRD)

The powder X-ray patterns for praziquantel with suberic samples were recorded in the angular range of 3 ≤ 2θ ≤ 40°, with a 0.02° step size and a scanning speed of 2°/min using monochromatic Cu Kα radiation (λ = 1.54056 Å) with a Shimadzu 6000 diffractometer (40 kV, 30 mA) equipped with a graphite monochromator and a Ni filter.

Room-temperature powder X-ray diffraction experiments for the remaining samples were performed using a Cu Kα radiation source on a D8 Advance Bruker AXS θ-2θ diffractometer (40 kV, 40 mA) equipped with a LYNXEYE-XE detector and a Ni filter. Data were collected in the 3 ≤ 2θ ≤ 60° range with a step size of 0.02°. Theoretical powder X-ray patterns, used to assess the purity of the obtained solids, were generated using Mercury software.^34^

### Single-Crystal X-ray Diffraction (SC-XRD)

Structural data for the **PZQ·SUB** single crystal were obtained based on the measurement performed on a SuperNova diffractometer equipped with dual X-ray microsources (Mo and Cu) and an Eos CCD detector with the X-ray tube operating at 50 kV and 0.8 mA. Data collection and data reduction, including Lorentz polarization and absorption effects, were performed using the CrysAlisPRO program package (CrysAlisPRO, Rigaku Oxford Diffraction, Yarnton, Oxfordshire, England, 2015). For the **PZQ·3HBA** (1:1) and **PZQ·BTC** cocrystals and for **PZQ·5HIP·MeCN** and **PZQ·TRI·H**_**2**_**O** cocrystal solvates, a Bruker AXS-KAPPA APEX II diffractometer with Mo Kα radiation and an X-ray generator operating at 50 kV and 30 mA was used for intensity data collection monitored by APEX3 software. The single-crystal diffraction data were then read in CrysAlisPRO and reduced. Each measurement was performed at room temperature. The crystal structures of PZQ systems were solved with SHELXT^35^ using intrinsic phasing. The structures were further refined via the SHELXL^36^ refinement package using least squares minimization, and all programs were implemented in the Olex2 program.^37,38^ The structure of **PZQ·TRI·H**_**2**_**O** was refined using a set of data collected to a resolution of *d* = 1.12 Å. H atoms were placed in idealized positions using the riding model and refined with fixed isotropic displacement parameters *U*_iso_(H) = 1.2*U*_eq_(C) for ternary CH and secondary CH_2_ groups and *U*_iso_(H) = 1.5*U*_eq_(O) for OH groups. Based on the difference Fourier map analysis, a noticeable position disorder in the cyclohexyl ring of the PZQ molecule in the **PZQ·SUB** structure was detected, successfully modeled, and refined considering 0.64 and 0.36 occupancy factors. Each carboxylic group in the BTC molecule in the **PZQ·BTC** cocrystal was disordered over two positions with fixed occupancies of 0.50. In the asymmetric unit of **PZQ·5HIP·MeCN**, solvent molecules could not be modeled satisfactorily; therefore, the solvent mask procedure implemented in Olex2 was applied. One of the water molecules in **PZQ·TRI·H**_**2**_**O** was disordered over two positions with fixed occupancies of 0.5.

### Differential Scanning Calorimetry (DSC) and Thermogravimetric Analysis (TG)

The thermal properties of the studied samples were analyzed with differential scanning calorimeter DSC-60 and DTG-60H equipment (Shimadzu Corporation). The instruments were calibrated with indium, and the reference sample was α alumina powder. From the DSC curves, the melting enthalpy, onset, and melting temperature were determined by TA-60 analysis software. The samples (3–4 mg) were placed in closed aluminum crucibles (Ø 6 mm × 1.5 mm) with perforated lids and heated from ambient temperature up to 200 °C, with a constant rate of 5 °C ·min^–1^ in a nitrogen atmosphere with a flow rate of 70 mL·min^–1^. The thermal decomposition temperatures and weight losses were obtained from TG measurements carried out from 25 up to 600 °C. The samples (7–8 mg) were placed in alumina crucibles (Ø 6 mm × 2.5 mm) and covered with a perforated lid in a nitrogen atmosphere with a flow rate of 70 mL·min^–1^.

### Fourier-Transform Infrared Spectroscopy (FT-IR)

A Jasco 6200 spectrometer was used to obtain the infrared absorption spectra, and the measurements were made in the wavelength range of 4000–400 cm^–1^, with a resolution of 4 cm^–1^ and 32 scans per spectrum. The spectra were obtained on pellets made from a mixture of approximately 1.5 mg of the analyzed samples with 200 mg of KBr. Spectral analysis was performed with SpectraManager software.

### X-ray Photoelectron Spectroscopy (XPS)

XPS measurements were performed with a SPECS PHOIBOS 150 MCD system equipped with a monochromatic Al Kα source (200 W, *h*ν = 1486.6 eV), a hemispherical analyzer, and a multichannel detector. The pressure of the vacuum in the analysis chamber during the measurements was in the range of 10^–9^ to 10^–10^ mbar, and charge neutralization was used for all samples. The binding energy scale was charge referenced to C 1s at 284.6 eV. Elemental compositions were determined from spectra acquired at a pass energy of 60 eV. High-resolution spectra were obtained using an analyzer pass energy of 20 eV, and the Shirley background subtraction method was used for the fitting procedure.

### Scanning Electron Microscopy (SEM)

The morphological analysis was performed with an FEI Quanta 3D FEG SEM microscope, in high vacuum, with an Everhart Thornley detector (ETD) at an acceleration voltage of 20 kV and magnification of 280× and 600× corresponding to 200 and 50 μm scale bars, respectively. The powdered samples (PZQ, SUB, and **PZQ·SUB**) were placed on a small piece of double-sided conductive carbon tape fixed to an aluminum stub and then sputter-coated with a thin film of gold (at a thickness of ∼10 nm) under an argon atmosphere to make them electrically conductive. The analysis was performed by comparing the morphology of the cocrystal with the morphology of the initial ingredients PZQ and SUB.

### Solubility Assessment by HPLC-UV Analysis

The solubility of the pure compound PZQ and its cocrystals using the shake-flask method was assessed using a 1200 series HPLC system (Agilent Technologies, USA) equipped with a DAD detector, employing a gradient mode elution (Table S3) at 1.2 mL/mL on a Waters Nova-Pak C18, 3.9 × 150 mm, 4 μm chromatographic column, maintained at 35 °C. The stock solution of PZQ (2 mg/mL) was prepared by dissolving the pure compound in MeCN. Corresponding aliquots were diluted with the same solvent for the preparation of working PZQ solutions. Samples of 10 μL were injected into the chromatographic system. Detection was performed at 210 nm, recording a 3.87 min retention time for the PZQ standard. For quantitative purposes, the peak areas of a PZQ calibration set (8 concentration levels) were used for linear regression (*y* = 33.763*x* + 154.99, 5–300 μg/mL PZQ). The hydro solubility of the parent compound and its cocrystals was assessed in three simulated biological fluids as dissolution media: the simulated gastric fluid (SGF) (0.144 mM CaCl_2_, 4.61 mM KH_2_PO_4_, and 34.56 mM NaCl, adjusted at pH 1.5), simulated intestinal fluid (SIF) (0.1 M NaHCO_3_, adjusted to pH 7.4), and simulated colonic fluid (SCF) (TRIS 16 mM buffer adjusted to pH 7.8). Ultrapure water was used as the reference dissolution medium. The solubility of the obtained PZQ cocrystals was determined by the shake-flask method. Briefly, excess PZQ and PZQ cocrystals (the corresponding amount of 6 mg of PZQ) were added to 2 mL vials containing 2 mL of the dissolution medium. The slurry samples were placed in an orbital shaker to achieve uniform mixing at 37 °C for 24 h. At different time intervals (10, 20, 30, 40, 50, 60, and 90 min), an aliquot of 200 μL of each sample was collected after centrifugation, filtered, and analyzed concerning the PZQ content by HPLC-UV analysis.

## Results and Discussion

### PXRD Analysis

For the mechanochemical synthesis of the cocrystals using 3HBA, 4HBA, VAN, OXA, and BTC as coformers, it was necessary to use acetonitrile to promote the supramolecular reactions. Experiments leading to the **PZQ·3HBA** cocrystals both in the 1:1 and 1:2 stoichiometric ratios (Figures S1 and S2), **PZQ·4HBA** 1:1 (Figure S3), and **PZQ·BTC** 2:1 (Figure S4) were conducted for 30 min. Praziquantel millings with VAN (Figure S5) and OXA (Figure S6) as coformers required reaction times of 45 and 60 min, respectively. The **PZQ·SAL·H**_**2**_**O** was obtained by acetonitrile-assisted grinding of the substrates for 30 min (Figure S7). Obtaining the **PZQ·TRI·H**_**2**_**O** 1:2:2 hydrate phase necessitated the use of praziquantel hemihydrate **PZQ·0.5H**_**2**_**O** as a substrate (Figure S8). It was obtained according to the procedure described in the paper of Perissutti et al.,^28^ consisting of preliminary neat grinding of praziquantel followed by the water addition and further grinding. As a next step, trimesic acid was added using different volumes of water. A pure phase was obtained by carrying out the reaction for 60 min with a volume of water corresponding to the wetting parameter η of 0.32 (Figure S9). The **PZQ·4ASA·MeCN** 1:1:1 (Figure S10) and **PZQ·5HIP·MeCN** 1:4:2 (Figure S11) cocrystal solvates were obtained by grinding with the addition of acetonitrile for 30 and 60 min, respectively. In the case of the samples **PZQ·SUB** 2:1 obtained by ball milling, in the presence of ethanol-dichloromethane, structural changes can be observed, beginning with the addition of the wetting liquid. The tests carried out in the preparation of PZQ samples with SUB for the same grinding frequency but different times and volumes of the solvent highlighted the appearance of a new form of **PZQ·SUB** when adding a minimum amount of the solvent (η of 0.78) and with a ball milling time of 30 min. The comparison of the powder patterns of the ball-milled samples with the diffractograms of the starting ingredients PZQ and SUB is presented in Figure S12.

### Single-Crystal Structure Description

From the samples obtained by the slow evaporation method, single crystals of suitable size for X-ray measurements were selected. The crystal structures of **PZQ·SUB**, **PZQ·3HBA 1:1**, **PZQ·BTC**, **PZQ·5HIP·MeCN**, and **PZQ·TRI·H**_**2**_**O** were elucidated by single-crystal X-ray diffraction measurement. Experimental and refinement details concerning the investigated crystal are presented in Tables S4 and S5.

### **PZQ·3HBA** 1:1 Cocrystal

**Figure 1:**
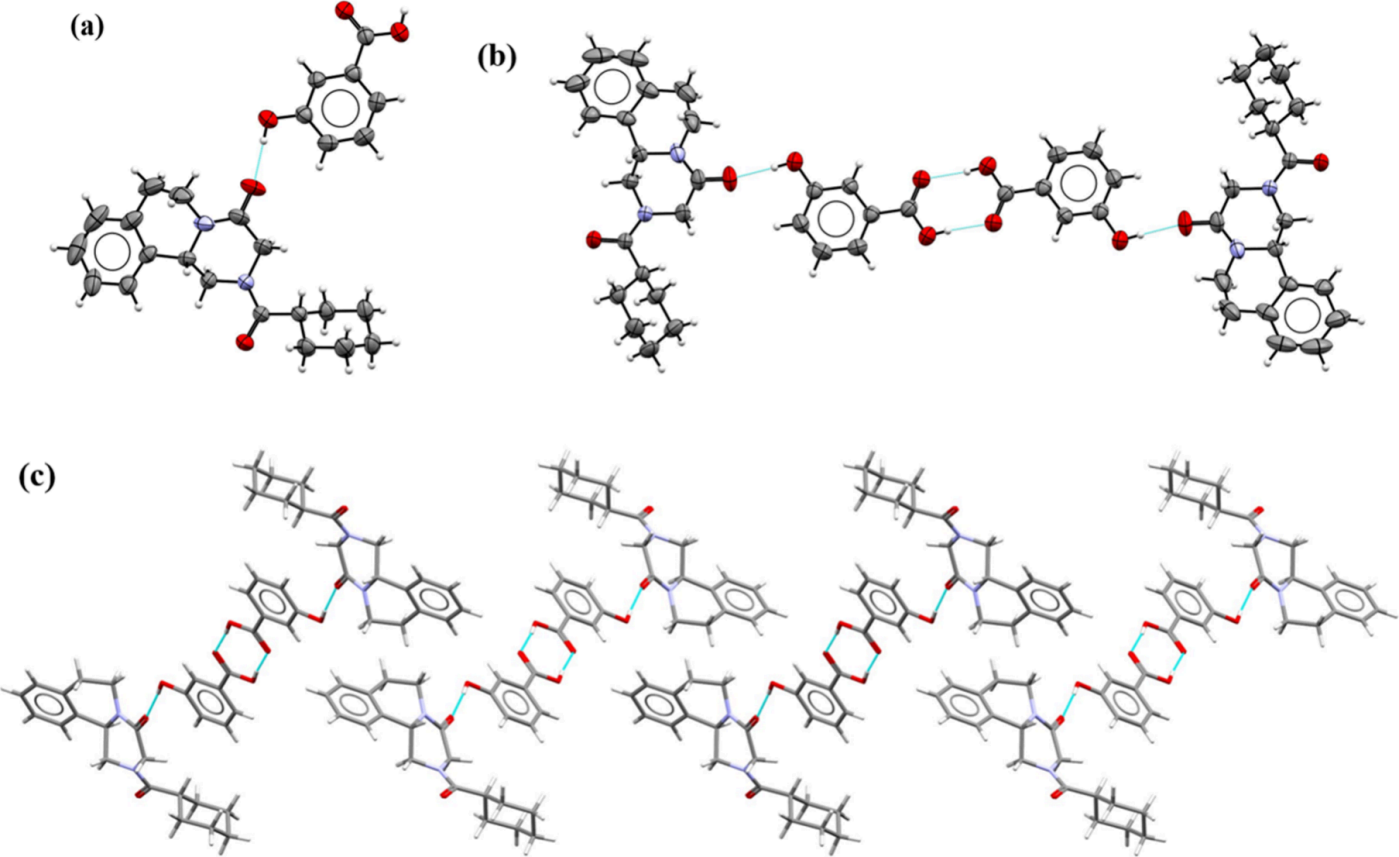
(a) ORTEP representation of the asymmetric unit of the **PZQ**·**3HBA** 1:1 cocrystal. Thermal ellipsoids were plotted at the 50% probability level. (b) ORTEP representation of the four-component system formed by O–H···O hydrogen bonds. (c) Fragment of crystal packing, in a view along the [010] direction.

PZQ with 3-hydroxybenzoic acid (3HBA) cocrystallize in a 2:1 stoichiometric ratio as a cocrystal in the *P*1̅ space group, which was published by Liu et al. in 2021.^32^ The studies presented in this work also showed the formation of a **PZQ·3HBA** cocrystal in a stoichiometric ratio of 1:1 (Figure 1a), crystallizing in the monoclinic space group *P*2_1_/*n*. In a crystal lattice, the centrosymmetric four-component motif formed by the O–H···O hydrogen bonds was observed (Figure 1b). In this system, the carboxyl group in the piperazinone fragment of the PZQ molecule acts in the O5–H5···O1 hydrogen bond as a proton acceptor from the hydroxyl group of the 3HBA molecule (Table S6). Based on the appropriate torsion angles, the *anti*-conformation of the praziquantel molecule can be confirmed (Table S6). The *R*_2_^2^(8) homosynthon formed by carboxylic groups of coformer molecules through O4–H4···O3^i^ noncovalent interactions was also formed. The 3D crystal structure is also stabilized by other interactions, like nonclassical C–H···O hydrogen bonds (Figure 1c).

### **PZQ·BTC** 2:1 Cocrystal

**Figure 2:**
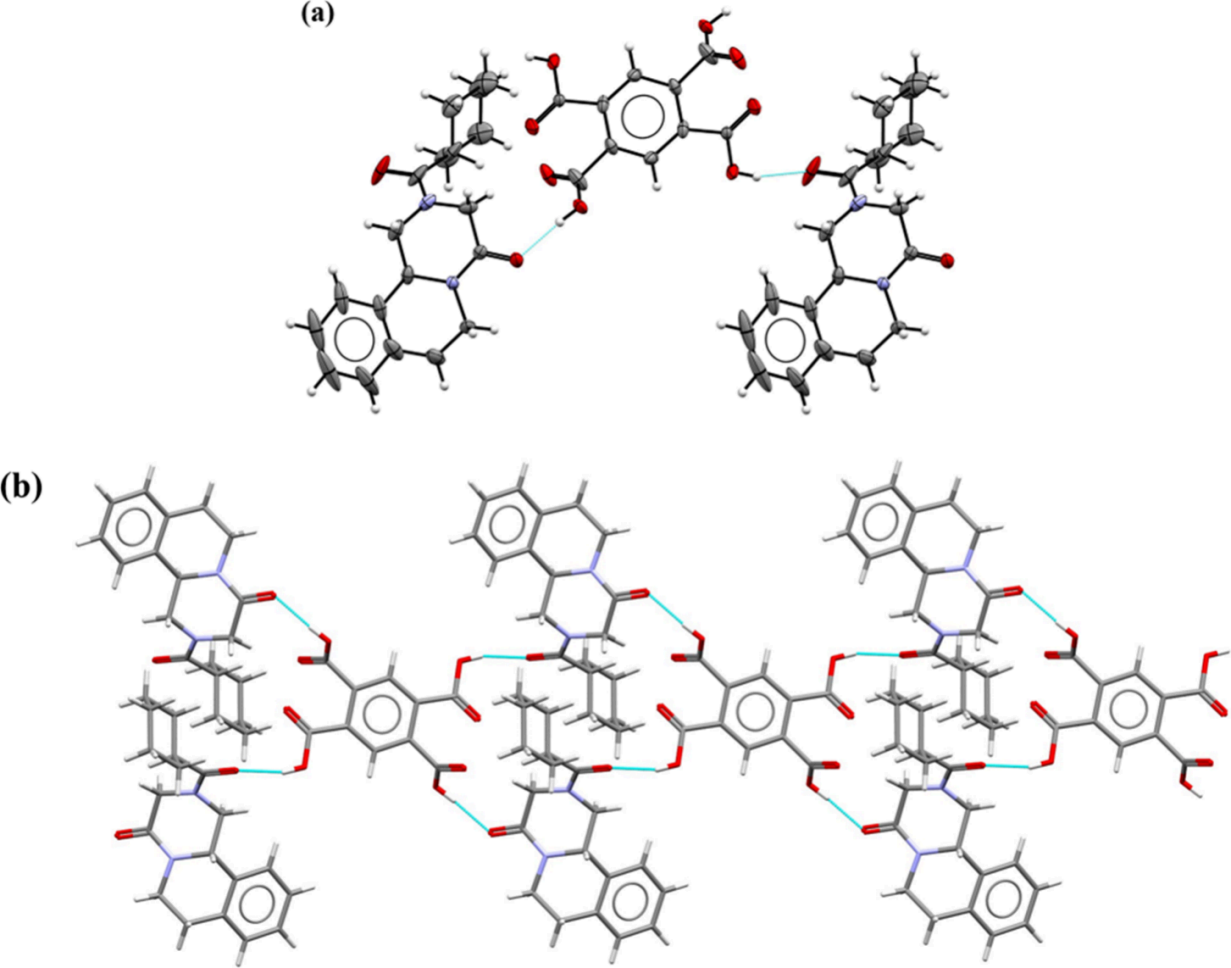
(a) ORTEP drawing of the **PZQ·BTC** 2:1 cocrystal fragment composed of two PZQ molecules and one BTC molecule. Thermal ellipsoids were plotted at the 30% probability level. (b) Intermolecular COOH···O=C interactions between PZQ and BTC components form a ribbon through the [011] direction, in a view along the [110] direction. Disordered fragments of the BTC molecule were omitted in the figures for clarity.

Praziquantel (PZQ) and benzene-1,2,4,5-tetracarboxylic acid (BTC) cocrystallize in the triclinic space group *P*1̅ with one PZQ molecule and half of the BTC molecule in the asymmetric unit. PZQ molecules in *anti*-stereochemistry (Table S7) are hydrogen-bonded with acid molecules via COOH···O=C interactions (Figure 2a), forming ribbons along the [011] direction (Figure 2b). Hydrogen bond details for **PZQ·BTC** 2:1 are given in Table S7. In this supramolecular motif, the *R*_4_^4^(28) synthons between the carboxylic moieties of BTC and C=O from PZQ are observed. The 3D crystal structure is also stabilized by C–H···O and π···π interactions.

### **PZQ·5HIP·MeCN** 1:4:2

**Figure 3:**
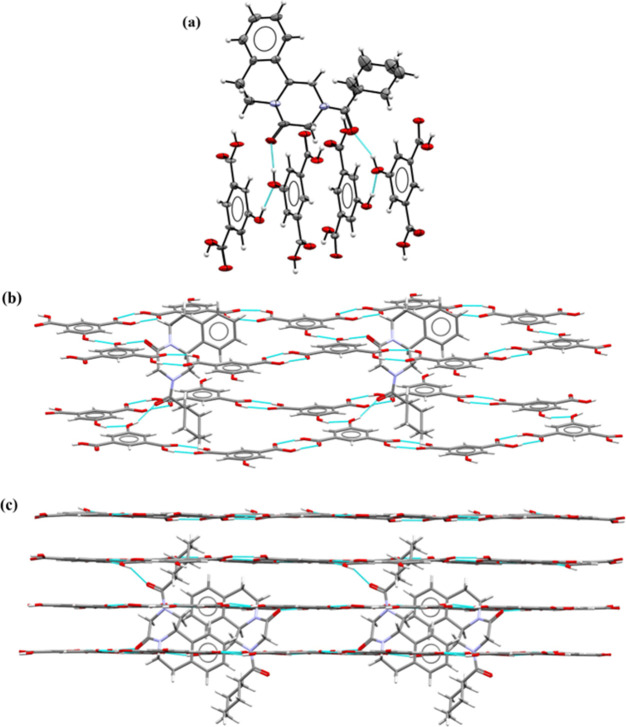
(a) ORTEP drawing of the **PZQ·5HIP·MeCN** 1:4:2 cocrystal solvate. Thermal ellipsoids were plotted at the 30% probability level. Solvent molecules have been omitted due to the solvent mask used. (b) Representation of the arrangement of acid molecules in layers in the hexagonal manner with PZQ molecules inside formed holes. (c) Arrangement of layers in the AB-AB-CD-CD order stabilized by stacking interactions between aromatic rings of 5HIP molecules.

Praziquantel (PZQ) and 5-hydroxyisophthalic acid (5HIP) cocrystallize as an acetonitrile cocrystal solvate in the triclinic space group *P*1̅ with one PZQ, four 5HIP molecules, and two acetonitrile molecules in an asymmetric unit (Figure 3a). A solvent mask was used due to difficulties in modeling solvent molecules. In the crystal structure, there are layers composed of 5HIP molecules connected by the O−H···O hydrogen bonds (Table S8) formed between carboxylic groups (homodimers *R*_2_^2^(8) composed of O–H_carboxyl_···O=C_carboxyl_) and hydroxyl groups (O–H_hydroxyl_···O–H_hydroxyl_ interactions), arranged in a hexagonal manner (Figure 3b). The formation of layers composed of noncovalently connected molecules A and B and another layer composed of molecules C and D is observed. These layers, arranged in the AB-AB-CD-CD-AB-AB-CD-CD order, are stabilized by π···π interactions between the aromatic rings of 5HIP molecules (Figure 3c). This way of arranging acid molecules results in the formation of holes in which praziquantel molecules are present. The maximum filling of the hole spaces with PZQ molecules was possible thanks to the adoption of the less preferred *syn*-conformation by the PZQ molecules, stabilized by the O–H_hydroxyl_···O=C_PZQ_ hydrogen bonds (Table S8).

### **PZQ·TRI·H_2_O** 1:2:2

**Figure 4:**
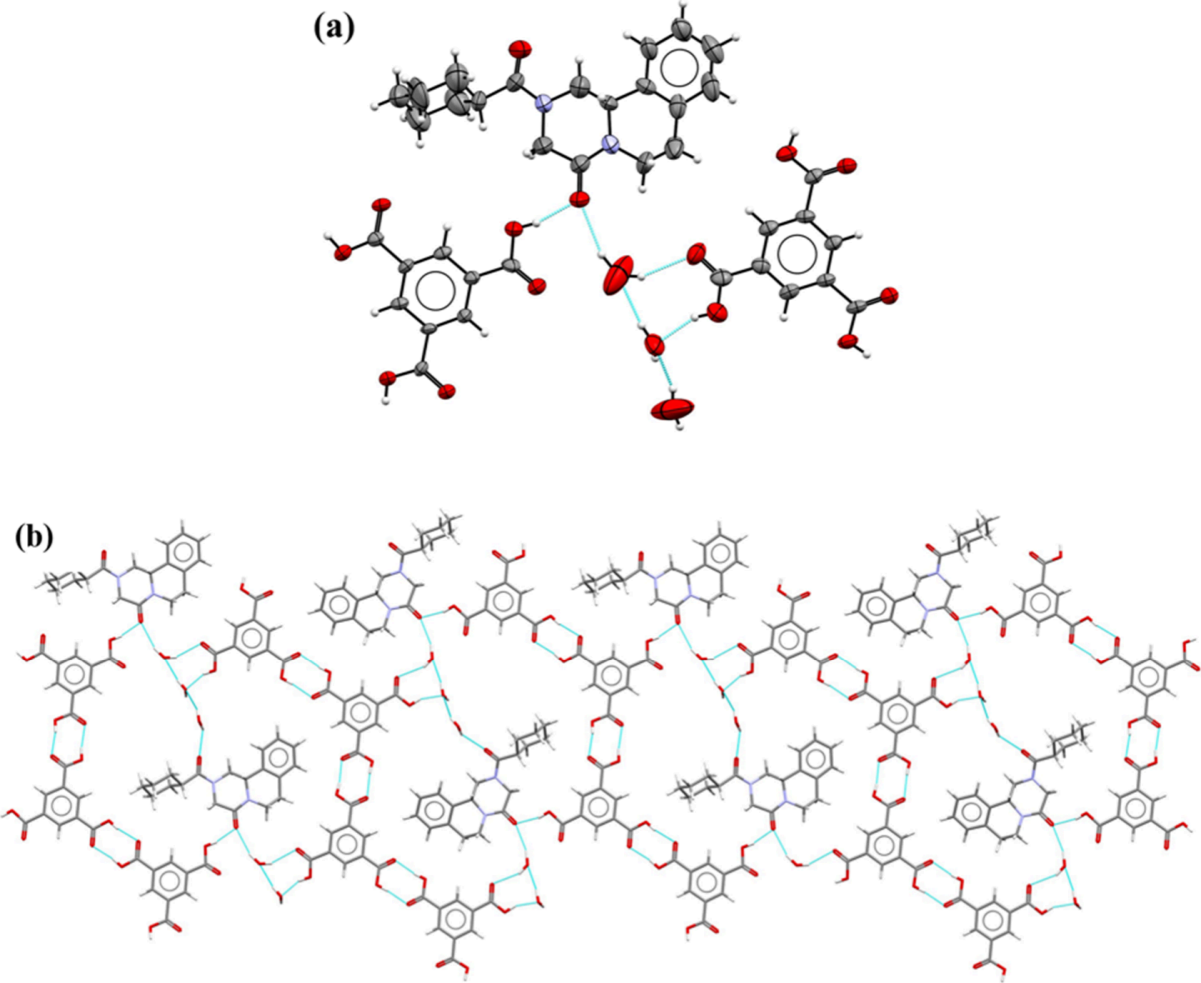
(a) ORTEP drawing of the asymmetric unit of **PZQ·TRI·H**_**2**_**O** at 1:2:2. Thermal ellipsoids were plotted at the 30% probability level. (b) Representation of intermolecular interactions between PZQ, TRI, and the water molecule in the **PZQ·TRI·H**_**2**_**O** 1:2:2 crystal lattice.

The asymmetric unit of the **PZQ·TRI·H**_**2**_**O** cocrystal solvate, which crystallizes in the monoclinic *C*2/*c* space group, consists of one PZQ, two TRI, and two water molecules (Figure 4a).

Praziquantel molecules are noncovalently connected to one of both trimesic acid molecules (labeled with A) through the O4A-H4AA···O1 hydrogen bond (Table S9). Both carbonyl groups of PZQ in the *anti*-conformation are hydrogen bond acceptors for two water molecules (O9–H9A···O1 and O10B–H10D···O2^v^ interactions). Carboxylic groups of trimesic acid molecules form *R*_2_^2^(8) synthons: one is formed through O6A-H6AA···O7A^iii^ and O8A-H8A···O5A^ii^ hydrogen bonds, and the second through O6B–H6BA···O7B^iv^ and O8B–H8B···O5B^i^ hydrogen bonds. One of the carboxyl groups is hydrogen-bonded with two water molecules (O9–H9B···O3B and O4B–H4BA···O10A interactions). There are also noncovalent interactions between water molecules (O10A-H10A···O9 and O10B–H10C···O10A hydrogen bonds). The supramolecular crystal packing is presented in Figure 4b, showing that the molecules are organized in layers in which these layers are connected by the water molecules.

### **PZQ·SUB** 2:1 Cocrystal

From the crystallographic data, it was determined that the **PZQ·SUB** cocrystal crystallizes in the monoclinic body-centered *I*2/*a* space group and was determined that the compound exists in a host:guest stoichiometric ratio of 2:1 (PZQ:SUB). The asymmetric unit consists of one praziquantel molecule and half of suberic acid (the other half being generated via 2/*a* symmetry operation since the suberic acid is sitting on an inversion center). The unit cell hosts eight praziquantel molecules and four suberic acid molecules. Similar molecular configurations with 2:1 host–guest stoichiometric ratios where the coformer (carboxylic acid) is located on inversion centers were previously reported in other multicomponent crystals (salts or cocrystals) of various active pharmaceutical ingredients such as etravirine^39^ or promethazine.^40^

**Figure 5:**
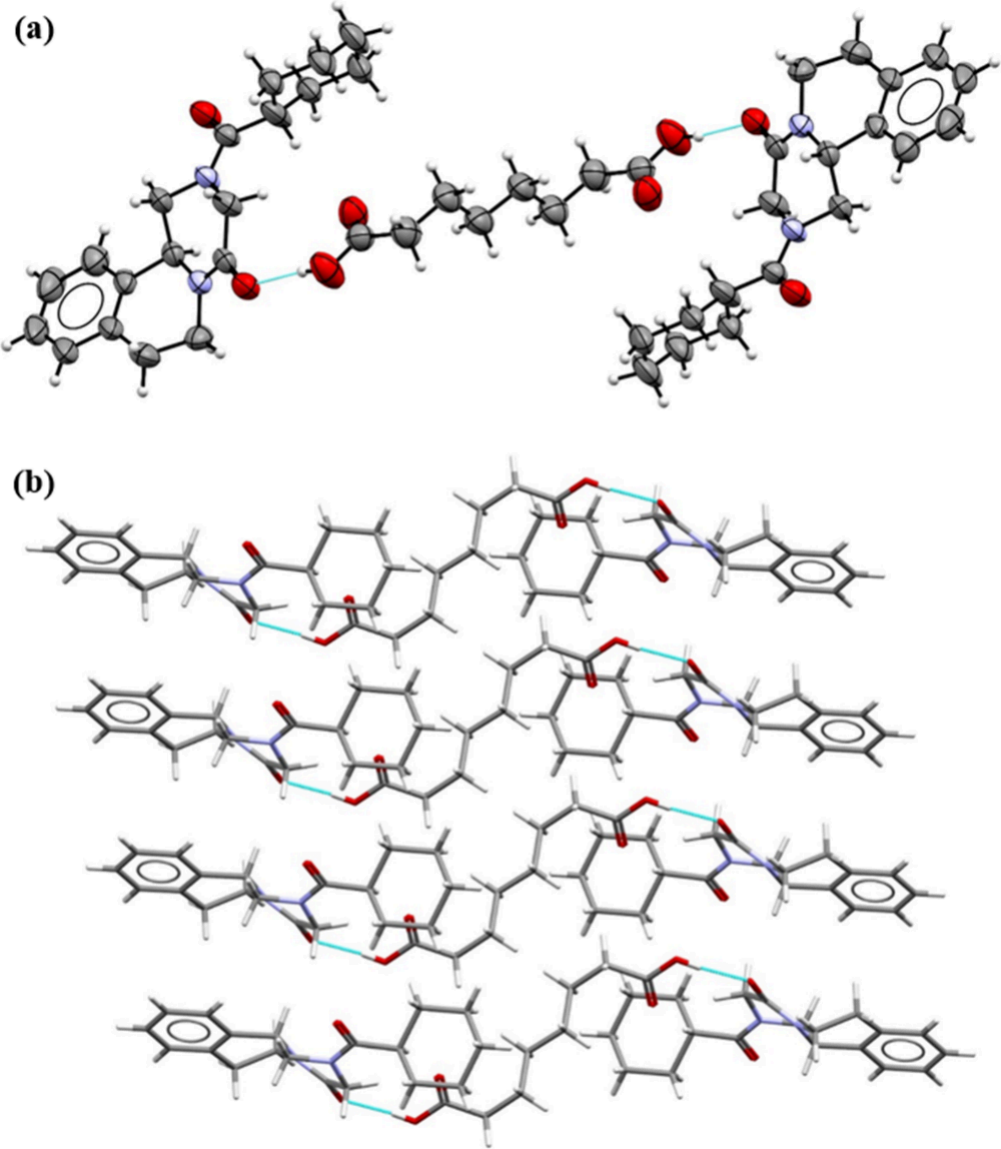
(a) ORTEP representation of the **PZQ-SUB** 2:1 cocrystal fragment with two PZQ molecules and one SUB molecule. Thermal ellipsoids were drawn at the 50% probability level. (b) Fragment of crystal packing for the **PZQ-SUB** 2:1 cocrystal.

The crystal **PZQ·SUB** can be described by molecular synthons, which consist of two praziquantel molecules and one suberic acid molecule bonded by hydroxyl···carbonyl O–H···O hydrogen bonding (Figure 5a). The supramolecular cohesion and the formation of 3D architectures are sustained by combinations of O–H···O, O–H···C, and C–H···O, which involved both carbonyl oxygen atoms of praziquantel molecules as acceptors and C–H···π interactions between the coformer and the phenyl ring of host molecules (Figure 5b). The intermolecular interactions with distances shorter than the sum of the van der Waals radii are listed in Table S10.

### Thermal Analyses and Physical Stability for **PZQ·SUB**

**Figure 6:**
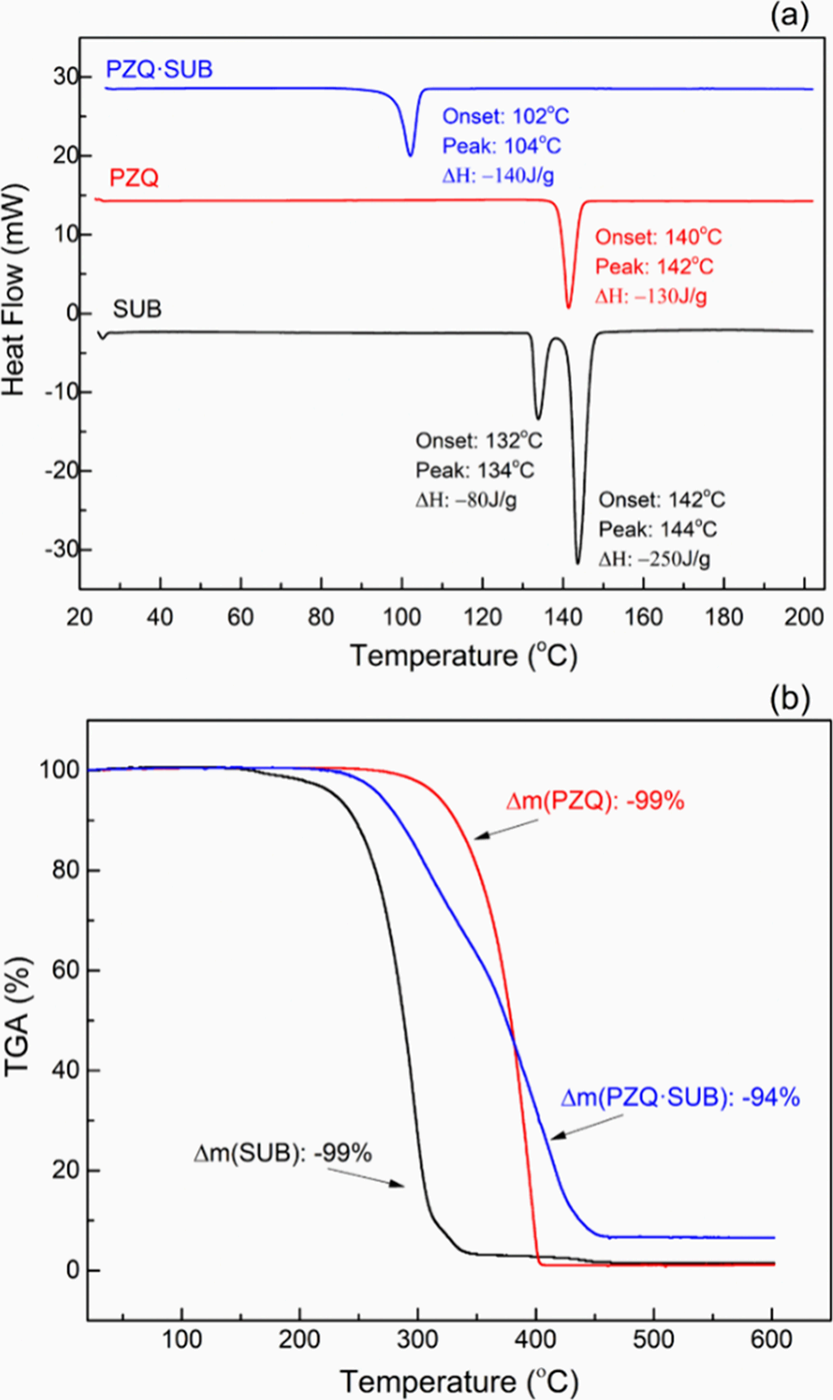
(a) DSC curves and (b) TGA thermograms.

The thermal properties of the starting compounds (PZQ and SUB) and the cocrystal (**PZQ·SUB**) were analyzed by differential thermal calorimetry (Figure 6a) and thermogravimetry (Figure 6b).

A single sharp endothermic peak characteristic of the melting of the substance is observed in the DSC curve of the cocrystal (Figure 6a). The temperature values at which the endothermic process begins (onset) and the maximum temperature of the signal (peak) are different compared to the values observed from the DSC curves of the pure ingredients. The melting of the cocrystal at lower temperatures compared to the pure components (PZQ and SUB) can be explained by the fact that the crystal lattice and the Gibbs energy of the cocrystal are lower than those of the individual components. The decrease in the melting point and enthalpy of fusion in the cocrystal compared to praziquantel alone indicates a decrease in the thermodynamic stability^41−44^ and implicitly a higher solubility.^45^ From the comparison of the TG curve of the cocrystal **PZQ·SUB** (Figure 6b) with the TG curves of the initial ingredients, it was observed that the decomposition of the cocrystal occurs more slowly compared to the initial ingredients, and the mass loss is lower. These results confirm that by cocrystallization of the compounds PZQ and SUB, the way of packing of the molecules involved in melting has changed, and the enthalpy calculated from the DSC curves has a higher value in the case of the cocrystal, compared to praziquantel alone.^45^

To verify the physical stability and if transformations of the cocrystal **PZQ·SUB** are occurring during storage for a long time, it was kept at 40 °C and an elevated humidity of 75% RH and was periodically measured by X-ray diffraction. The results showed great stability of this cocrystal for a period of up to 5 months (Figure S13a). An interesting effect was observed in the case of the physical mixture of PZQ with SUB obtained in the sample obtained by dry milling for 30 min. After 40 days of storage in the climatic environment with controlled humidity and temperature, the ingredients established intermolecular bonds, so that after 100 days, the physical mixture has transformed into the cocrystal **PZQ·SUB**. This result highlights that the cocrystal could be obtained if a physical mixture of PZQ and SUB is stored for 100 days in a high-temperature and high-humidity environment without the need for solvent wetting (Figure S13b).

### FT-IR Results for the **PZQ·SUB** 2:1 Cocrystal

**Figure 7:**
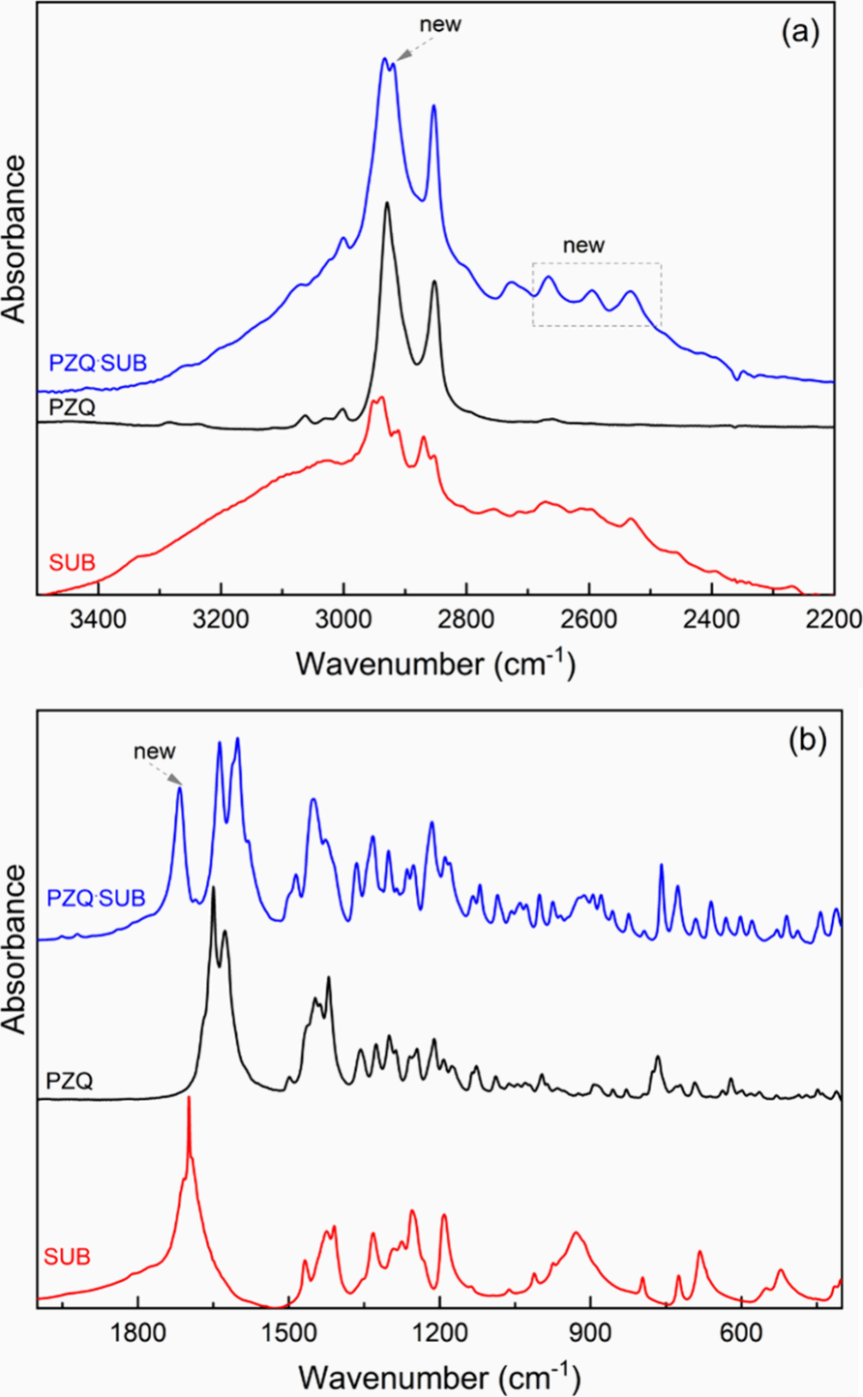
FT-IR spectra of SUB, PZQ, and **PZQ·SUB** in the spectral ranges of (a) 3500–2200 and (b) 2000–400 cm^–1^.

From the comparison of the infrared spectrum obtained on the new **PZQ·SUB** cocrystal with the spectra of the initial ingredients (**SUB** and **PZQ**), it was observed that changes occur in the vibrational frequency values (Figure 7). In the **PZQ·SUB** cocrystal, the two oxygen atoms of the carbonyl group (C=O) in PZQ acted as hydrogen bond acceptors, and the hydrogen atoms of the carboxyl groups in suberic acid were hydrogen bond donors.

The characteristic functional groups observed in the spectrum of **PZQ** are as follows: −CH, −CH_2_, and −CH_3_ stretching (one intense band at 2928 cm^–1^ and another of medium intensity at 2852 cm^–1^), carbonyl stretch vibrations −C=O (1630 cm^–1^), and −C–N stretching (1000–1350 cm^–1^).

The characteristic vibration in SUB spectra appeared between 3200 and 3500 cm^–1^ due to the hydroxyl stretching vibration −OH and between 1700 and 1800 cm^–1^ due to the carbonyl −C=O stretching vibration. In the **PZQ·SUB** cocrystal spectrum between the 2700 and 2500 cm^–1^ region, new bands of low intensity appear due to hydrogen bonds established between praziquantel and suberic acid.

The characteristic band of the vibration of the −OH bond from 2928 cm^–1^ in PZQ decreases in intensity in the cocrystal spectrum and is shifted to 2933 cm^–1^; this change indicates that the hydroxyl group CHOH from suberic acid participates in the formation of the hydrogen bond with the carbonyl groups from PZQ. Weak new bands appear at 2920, 2726, 2666, 2595, and 2532 cm^–1^ due to the O–H stretching hydrogen-bonded interactions (Figure 7a).

The intermolecular interactions between PZQ and SUB are best observed in the spectral region of 1800–1100 cm^–1^ (Figure 7b). Thus, in the spectrum of the **PZQ·SUB** sample, the characteristic bands of the carbonyl stretching vibrations of the two groups (C=O/amide I),^46^ located at 1650 and 1627 cm^–1^ in the PZQ spectrum, appear at low vibration frequencies, at 1638 and 1601 cm^–1^, respectively. These changes indicate the cocrystal formation by intermolecular bonds.^46^ In addition, the −C=O stretching vibration present in suberic acid at 1699 cm^–1^ is shifted to higher wavenumbers in the cocrystal, at 1715 cm^–1^.^23^ This displacement of the C=O vibration occurs when the −OH of the carboxyl group in suberic acid forms intermolecular bonds with praziquantel, thus reducing the strength of the intramolecular hydrogen bonds between C=O and −OH. The ball mill process applied to praziquantel and suberic acid produces significant changes in the intensities and positions of the bands located in the 1800–1100 cm^–1^ spectral region. The displacement of the amide I band toward lower frequency values and the new vibration bands are clear proof of the formation of a new praziquantel cocrystal with suberic acid.

### XPS Analysis for **PZQ·SUB**

The XPS technique is frequently used to characterize the surfaces of materials because it can provide complete and highly accurate information about the chemical composition and oxidation state of the identified chemical elements. Furthermore, since XPS is sensitive to the degree of proton transfer, it has been shown that it can be very useful in distinguishing between new forms, i.e., a cocrystal or a salt, of active pharmaceutical ingredients just by analyzing the binding energy value of the N 1s photoelectron peak.^47^ It is known that the formation of cocrystals between an active pharmaceutical ingredient and a coformer is an assembly of the components through intermolecular interactions, including hydrogen bonds,^48^ while in the formation of a salt, protonation occurs between the active pharmaceutical ingredient and the coformer used.^47,49,50^ Upon obtaining a new form, the shift of the XPS N 1s peak toward higher binding energy values (around 402 eV) is due to the protonation of nitrogen and thus indicates the formation of a salt, while in the case of a cocrystal, the absence of this peak reveals the lack of protonation.^51^ In this sense, for a good characterization of the samples, both survey and C 1s, N 1s, and O 1s high-resolution spectra for each identified chemical element were recorded and then carefully analyzed.

The survey spectra of PZQ and **PZQ·SUB** samples (Figure S14a) show the peaks related to C, O, and N, while for the SUB sample, the peaks of C and O are present; thus, the chemical composition of the sample was confirmed. The presence of other elements that could indicate contamination of the samples was not highlighted.

The theoretical relative atomic concentration calculated from the chemical formula and the experimental one resulting from the analysis of the survey spectra for all samples are listed in Table 1.

**Table 1:** Theoretical and Experimental Relative Atomic Concentrations (at. %) of Chemical Elements Calculated from the Chemical Formula and XPS Survey Spectra, Respectively

		**relative atomic concentration** (at. %)							
		experimental			theoretical			experimental	theoretical
**sample**	**chemical formula**	O	C	N	O	C	N	C/O	C/O
**PZQ·SUB**	2*C_19_H_24_N_2_O_2_: C_8_H_14_O_4_	13.2	81.4	5.4	13.8	79.3	6.9	6.17	5.75
PZQ	C_19_H_24_N_2_O_2_	9.3	83.2	7.5	8.7	82.6	8.7	8.95	9.5
SUB	C_8_H_14_O_4_	26.9	73.1		33.3	66.7		2.75	2

By XPS analysis, it is not possible to detect hydrogen because its 1s photoelectron has a very small cross section for photoemission. Therefore, when calculating the theoretical atomic concentrations, the hydrogen concentration was also excluded for similarity. First, very important to observe is that the experimental at. % values obtained from the spectral analysis are very close to the theoretical ones for all the samples. Small differences in the concentration of carbon or oxygen can be attributed to adventitious contamination species of the sample surfaces during atmospheric exposure. The elemental composition of the **PZQ·SUB** sample (Table 1) determined from the survey spectra shows that the C, O, and atomic N percentages are close to those expected for **PZQ:SUB** = 2:1 stoichiometry as a result of the XRD analysis.

The deconvolution of C 1s spectra can provide valuable information on the presence and number of functional groups, both nonoxygen-containing groups such as C–C, C=C, C–H, or C–N as well as oxygen-containing groups including carbonyl (C=O) and carboxyl (O=C–OH).^47^

The high-resolution spectra for each identified chemical element were recorded and then carefully analyzed.

The C 1s high-resolution spectra recorded for SUB, PZQ, and **PZQ·SUB** samples show asymmetry toward high binding energies (Figure S14b).

**Figure 8:**
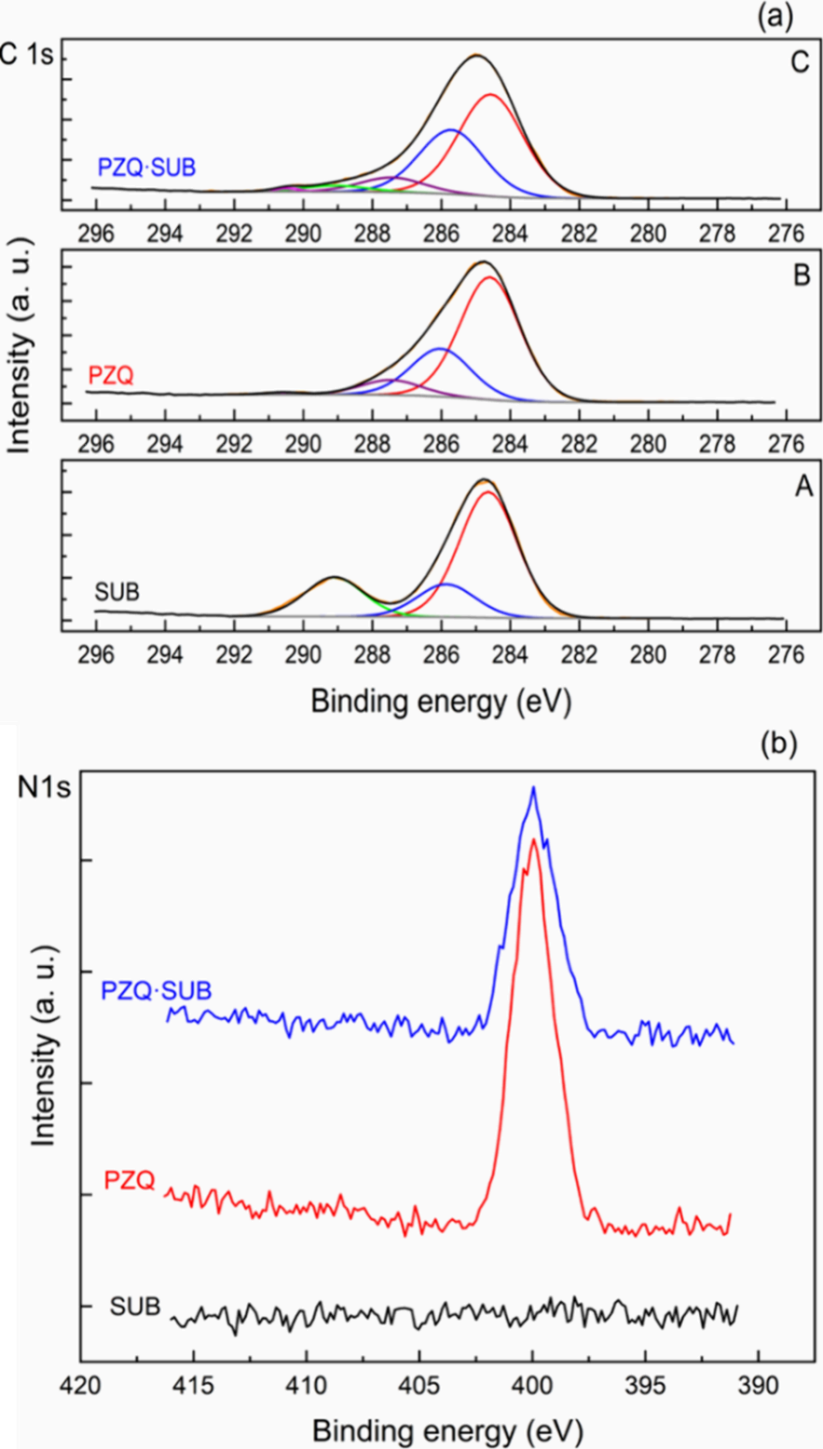
(a) C 1s deconvolution spectra and (b) N 1s high-resolution spectra of PZQ, SUB, and **PZQ·SUB**.

Therefore, the deconvolution of these spectra could be done, considering the structure and composition of the samples, using three components for SUB, four components for PZQ, and five components for the **PZQ·SUB** sample, corresponding to different chemical environments of carbon atoms. The results obtained from the deconvolution of C 1s XPS spectra for all the samples are presented in Figure 8a and are further summarized in Table 2.

**Table 2:** Fraction (*f*) of Carbon Atoms Involved in Different Bonds Determined from Deconvolution of the XPS C 1s Core-Level Spectra

	**C bonds**									
	**C–C, **C–H****		C****–**N, C–OH, C****–**COOH****(53)		**C=O, **carbonyl group, N**–****C=**O****(55)		**O=C–OH, **carboxyl group****(53)		π**–**π*	
**sample**	**BE (eV)**	***f* (%)**	**BE (eV)**	***f* (%)**	**BE (eV)**	***f* (%)**	**BE (eV)**	***f* (%)**	**BE (eV)**	***f* (%)**
**PZQ·SUB**	284.6	54	285.8	34	287.4	7.9	289	2.7	290.5	0.7
**PZQ**	284.6	65.5	286	25.7	287.5	8			290.5	0.8
**SUB**	284.6	63.5	285.9	16.6			289.1	19.9		

The C 1s spectrum for the SUB sample (Figure S14b) can be resolved into three components at 284.6, 285.9, and 289.1 eV corresponding to carbon–carbon and carbon–hydrogen bonds C–C/C–H, to carbon linked to the adjacent carboxyl group C–COOH (the second neighbor interaction chemical shift),^50,52,53^ which can overlap with contamination species C–OH, and the third to carboxylate groups O–C=OH, respectively.^47,54^

Analysis of the C 1s spectrum of the PZQ sample (Figure S14b) is more complex because of several types of carbon environments. The deconvolution of the PZQ C 1s core-level spectrum (Figure 8a) reveals four components with binding energies at 284.6, 286, 287.5, and 290.5 eV. The first two components at lower binding energies are assigned to C–C/C–H bonds and structural carbon to nitrogen C–N bonds superposed probably with C–OH bonds from atmospherically contaminated species.^56^ The peak at 287.5 eV corresponds to C=O carbonyl groups and N–C=O amide carbon bonds.^52^ The last component at 290.5 eV is assigned to the π–π* shakeup satellite indicating the presence of aromatic rings and the sp^2^ state of carbon.^57^

The C 1s spectrum for the **PZQ·SUB** sample can be resolved into five components located at binding energy values that indicate the presence of functional groups specific to the two starting compounds, namely, PZQ and SUB, in the form of this new compound. As previously discussed, for all the samples, the dominant component peak located at 284.6 eV can be assigned to C–C and C–H bonds. The peak at 285.8 eV can be related to C–N bonds from the PZQ compound and at the same time to C–COOH^53^ of the SUB molecule hydrogen-bonded to two PZQ molecules as XRD revealed. The components at 287.4 and 289 eV could be related to C=O and N–C=O^52^ in PZQ and O=C–OH groups of suberic acid,^50,53,54^ respectively. The last component at 290.5 eV could be the π–π* shakeup satellite coming from aromatic rings of PZQ molecules.^57^

The high-resolution N 1s XPS spectra of PZQ and **PZQ·SUB** samples (Figure 8b) are symmetrical, indicating one type of binding configuration related to the involved nitrogen atoms. The binding energy of N 1s photoelectrons can make an unequivocal distinction between the protonated nitrogen species specific to salt formation and the hydrogen-bonded nitrogen species involved in the cocrystal structure. The absence of the N 1s peak component at a higher binding energy around 402 eV for the **PZQ·SUB** sample revealed an unprotonated nitrogen atom and hence the cocrystal formation.^47,51^ The N 1s spectra for PZQ and **PZQ·SUB** samples can be well-fitted with one component at around 400 eV assigned to sp^2^-hybridized N atoms that are bonded to three C atoms (e.g., C–N(−C)–C)^58^ and C–N/N–C=O nitrogen environments.^47,56^

### SEM for **PZQ·SUB**

**Figure 9:**
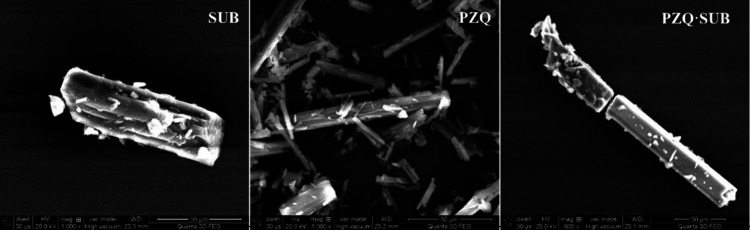
SEM micrographs of the starting ingredients (SUB and PZQ) and of the cocrystal of praziquantel/suberic acid (**PZQ·SUB**), at 1000× magnification, zoom 801/738, and scale bars of 50 μm.

The SEM micrographs at different magnifications, obtained for the starting ingredients (PZQ and SUB) and on the new cocrystal **PZQ·SUB**, are presented in Figure 9.

The SEM images of suberic acid (SUB) show a prismatic appearance with thin sheets, about 100 μm in length, compared with the SEM images of praziquantel (PZQ), which have a parallelepiped appearance of about 20–100 μm in length. The morphology of the **PZQ·SUB** cocrystal is distinguishable from the singular or mixture of the ingredients PZQ or SUB. The cocrystal has a shape-layered structure of thin parallelepiped sheets of about 100–300 μm in length.

### Solubility Assessment by the Shake-Flask Method

**Figure 10:**
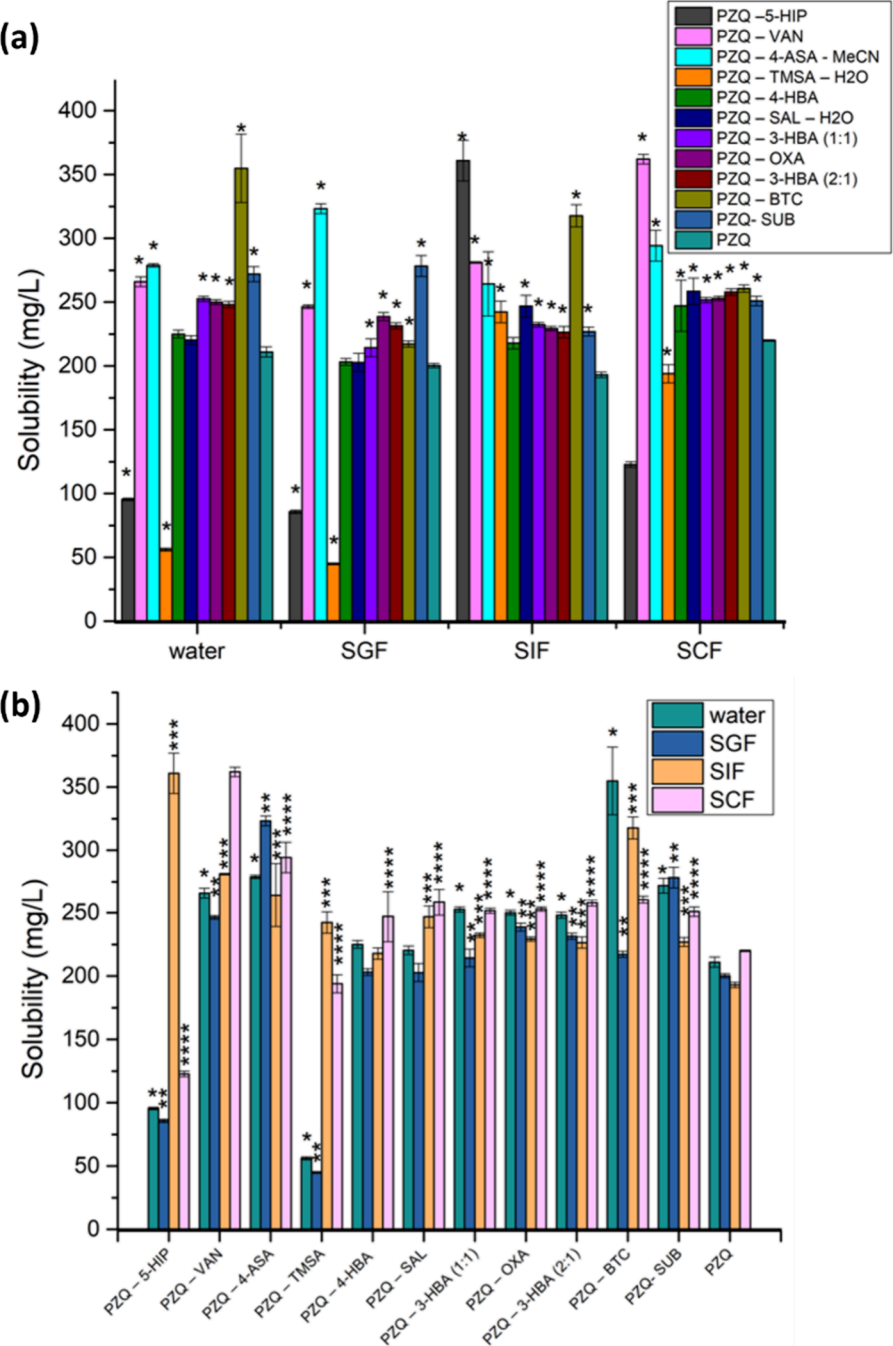
(a,b) Average equilibrium solubilities (*n* = 3) of studied PZQ cocrystals in different dissolution media.

The solubilities of each cocrystal and PZQ were tested in ultrapure water and the three dissolution media, simulating the biorelevant fluids present along the entire gastrointestinal tract, namely, SGF (simulated gastric fluid), SIF (simulated intestinal fluid), and SCF (simulated colonic fluid). The levels of PZQ attained in the studied dissolution media using the shake-flask method are depicted in Figure 10 and Table S11.

This piece of data is important for understanding the solubility characteristics of praziquantel cocrystals in different physiological conditions, which is crucial for the future development of effective drug formulations and dosage forms. The equilibrium solubility of praziquantel at 24 h strongly varies across the different cocrystals and dissolution media. The selection of a unique candidate that performs best under all tested conditions is less straightforward.

As a typical BCS class II representative, PZQ absorption mainly occurs within the small intestine, specifically from the duodenum and ileum, due to its high permeability and high surface area and much less from the colon and stomach. Additionally, the short transit time through the stomach and its thick mucus layer typically limit drug absorption.^59,60^

Therefore, SIF may be considered the most relevant media for comparing solubility data for the newly synthesized cocrystals. Figure 10a shows the highest solubility in SIF for **PZQ·5HIP·MeCN** of 361.00 μg/mL, 1.87-fold higher than PZQ alone (193.04 μg/mL). However, **PZQ·5HIP·MeCN** had significantly lower solubility in SGF (85.70 μg/mL) and SCF (122.86 μg/mL), even compared to PZQ alone (200.30 μg/mL in SGF and 220.01 μg/mL in SCF), suggesting that **PZQ·5HIP·MeCN** might not be the best candidate since the lower solubility in SCF and SGF could potentially limit overall bioavailability.

**PZQ·BTC** showed the second best solubility in SIF (317.60 μg/mL) and was superior in the other media (217.19 μg/mL in SGF and 260.75 μg/mL in SCF), compared to both **PZQ·5HIP·MeCN** and PZQ alone. Due to its enhanced solubility throughout the different segments of the gastrointestinal tract, **PZQ·BTC** seems to be the most promising candidate for higher absorption and increased oral bioavailability.

For a better overview of the whole data set, principal component analysis (PCA) has been performed considering the studied cocrystals’ equilibrium solubility data in all dissolution media (water, SGF, SIF, and SCF) as variables. The unsupervised multivariate data analysis tool revealed the cocrystals with an average solubility profile, closer to the origin in the model’s biplot (1PC explaining 64.8% of the total variability), in line with one of the parent compounds, PZQ (Figure S15A). **PZQ·4ASA·MeCN**, **PZQ·VAN**, and to a lesser degree **PZQ·BTC** and **PZQ·SUB** stand out in terms of their superior solubility in water as well as in simulated fluids of the upper (SGF) and lower (SCF) GI tract. On the other hand, **PZQ·5HIP·MeCN** and **PZQ·TRI·H**_**2**_**O** are best performing in SIF. The trends are slightly changing if only SIF and SCF are kept as predictor variables (Figure S15B), with the most promising cocrystals in terms of enhanced hydrosolubility in the GI tract and potentially improved oral bioavailability in comparison with pure PZQ being cocrystals furthest from the origin, found in the upper left and right quadrants of the PCA biplot, namely, **PZQ·VAN**, **PZQ·BTC**, and **PZQ·5HIP·MeCN**.

A dynamic solubility assay covering the initial phase of the process with multiple sampling points (10, 20, 40, 60, and 90 min) for PZQ and selected cocrystals using the shake-flask method combined with chromatographic analysis in the three simulated biological environments was also performed (data not shown). However, for all tested cocrystals close to PZQ, equilibrium concentrations (24 h) were achieved within the first sampling point (10 min). Considering the ideal case of a fast disintegration of the orally administered cocrystal-based solid pharmaceutical formulation, such a solubility profile would favor higher rates of PZQ absorption as it travels through the gastrointestinal tract, counteracting to some extent the effects of an extensive first-pass metabolization.^60^

In conclusion, the equilibrium solubility assay showed statistically significant enhancements in most cases of the studied cocrystals and all biorelevant dissolution media in comparison with the pure parent compound. Nevertheless, in addition to the already reported **PZQ·VAN**, the most promising perspectives in terms of the overall oral bioavailability are expected for the cocrystals of PZQ with BTC and 5HIP among the newly synthesized cocrystals. **PZQ·SUB** demonstrated noteworthy solubility in comparison with pure PZQ, performing second best in SGF and water.

## Conclusions

The mechanochemical synthesis of new praziquantel cocrystals with generally recognized as safe (GRAS) coformers represents a promising approach in pharmaceutical research. Systematic experimentation and analysis demonstrated that it is possible to continue exploring mechanochemistry to generate novel cocrystals of praziquantel. This study provides valuable insights into the impact of mechanochemical conditions on the formation and stability of praziquantel cocrystals, particularly the influence of the solvent on LAG processes. The cocrystal physicochemical characterization, including their crystal structures, thermal properties, and spectroscopic features, adds significant knowledge to the understanding of the newly formed compounds.

Enhancing drug solubility through the cocrystallization approach is paramount in pharmaceutical research and development due to its ability to address solubility challenges, thereby potentially improving drug bioavailability, efficacy, and patient compliance, ultimately advancing the therapeutic landscape. Most of the praziquantel cocrystals and all biorelevant dissolution media studied herein proved to have the ability to change praziquantel’s solubility. In addition to the already reported **PZQ·VAN**, the most promising results in terms of overall oral bioavailability are expected for the cocrystals of PZQ with BTC and 5HIP; **PZQ·SUB** demonstrated noteworthy solubility in comparison with pure PZQ, performing second best in SGF and water. The **PZQ·BTC** cocrystal has shown to be one of the most promising regarding solubility.

In summary, this study expands the understanding of mechanochemistry while presenting alternatives for the development of more soluble and therefore effective formulations of the anthelmintic compound praziquantel.

## Supporting Information Available

Experimental details, including the ball milling synthesis, PXRD patterns, crystallographic data, physical stability, XPS, and HPLC analyses (PDF)
