# Supplementary material for: Mechanochemical Synthesis of New Praziquantel Cocrystals: Solid-State Characterization and Solubility
Source: Cryst Growth Des. 2024 May 11;24(11):4668–81. doi: 10.1021/acs.cgd.4c00296 (PMC11157481; doi:10.1021/acs.cgd.4c00296)
Supplement: Supplementary file 1 — cg4c00296_si_001.pdf [file cg4c00296_si_001.pdf]

Supplementary information

Mechanochemical synthesis of new Praziquantel co-crystals: Solid-State Characterization and solubility

Marieta Mureșan-Pop,[1,2*] Simion Simon,[1,2] Ede Bodoki,[3] Viorica Simon,[1] Alexandru Turza[4], Milica Todea,[1,2,5] Adriana Vulpoi,[1,2] Klara Magyari,[1] Bogdan-C. Iacob,[3] Alexandra Iulia Bărăian,[3] Mateusz Gołdyn,[6,7] Clara S. B. Gomes,[7] Margarida Susana,[9] M. Teresa Duarte,[9] Vânia André[9,10*]

[1] Nanostructured Materials and Bio-Nano Interfaces Department, Interdisciplinary Research Institute on Bio-Nano-Sciences, Babes-Bolyai University, 42, Treboniu Laurian, 400271, Cluj-Napoca, Romania

[2] INSPIRE Research Platform, Babes-Bolyai University, 11, Arany Janos, 400028 Cluj-Napoca, Romania

[3] Analytical Chemistry Department, Faculty of Pharmacy, Iuliu Hațieganu University of Medicine and Pharmacy, 4, Louis Pasteur, 400349, Cluj-Napoca, Romania

[4] Mass Spectrometry, Chromatography and Applied Physics Department, National Institute for Research and Development of Isotopic and Molecular Technologies 400293, Cluj-Napoca, Romania

[5] Molecular Sciences Department, Faculty of Medicine, Iuliu Hațieganu University of Medicine and Pharmacy, 4, Louis Pasteur, 400349, Cluj-Napoca, Romania

[6] Faculty of Chemistry, Adam Mickiewicz University in Poznań, Uniwersytetu Poznańskiego 8, Poznań 61-614, Poland

[7] Center for Advanced Technology, Adam Mickiewicz University in Poznań, Uniwersytetu Poznańskiego 10, Poznań 61-614, Poland

[8] LAQV-REQUIMTE, Department of Chemistry, NOVA School of Science and Technology

(NOVA FCT), NOVA University of Lisbon, 2829-516 Caparica, Portugal

[9] Centro de Química Estrutural, Institute of Molecular Sciences, Instituto Superior Técnico, Universidade de Lisboa, Av. Rovisco Pais, 1049-001 Lisboa, Portugal

[10] Associação do Instituto Superior Técnico para a Investigação e Desenvolvimento (IST-ID), Avenida António José de Almeida, 12, 1000-043 Lisboa, Portugal

TABLE OF CONTENTS

1. **Ball milling synthesis**
2. **Powder X-ray diffraction**
3. **Crystallographic details**
4. **Physical stability of PZQ·SUB co-crystal, and PZQ·SUB physical mixture**
5. **XPS details for PZQ·SUB**
6. **Solubility details**

1. Ball milling synthesis

Table S1. Details regarding the mechanochemical synthesis conditions – PZQ/coformer stoichiometry, type and volume of solvent, parameter η value, and grinding time.

Co-crystal former	Stoichiometry PZQ/cocrystal former	Solvent (volume)	η parameter / µL·mg^{-1}	Reaction time (min)
**salicylic acid (SAL)**	1:1:1 [a]	CH_3CN (40 µl)	0.16	30
**3-hydroxybenzoic acid (3HBA)**	2:1	CH_3CN or C_2H_6O (40 µl)	0.16	30
	1:1	CH_3CN (40 µl)	0.16	30
**4-hydroxybenzoic acid (4HBA)**	1:1	CH_3CN (40 µl)	0.16	30
**4-aminosalicylic acid (4ASA)**	1:1:1 [b]	CH_3CN (40 µl)	0.16	30
**vanillic acid (VAN)**	1:1	CH_3CN (40 µl)	0.16	45
**oxalic acid (OXA)**	1:1	CH_3CN (40 µl)	0.16	60
**trimesic acid (TRI)**	1:2:2 [c]	H_2O (80 µl)	0.32	60
**benzene-1,2,4,5-tetracarboxylic acid (BTC)**	2:1	CH_3CN (40 µl)	0.16	30
**5-hydroxyisophthalic acid (5HIP)**	1:4	CH_3CN (40 µl)	0.16	60
**suberic acid (SUB)**	2:1	no solvent (neat grinding)	0	30 / 60
		C_6H_6O/CH_2Cl_2 (200 µl)	0.78	30 / 60

		C_6H_6O/CH_2Cl_2 (400 µl)	1.56	30 / 60

a) cocrystal hydrate; b) cocrystal solvate with acetonitrile; c) PZQ·0.5H_2O obtained by earlier PZQ grinding with water was used for grinding with trimesic acid (B. Perissutti paper, 2020, 10.3390/pharmaceutics12030289)

Table S2. Amount of PZQ and coformer used for grinding.

**Sample**	**n_{PZQ} (mmol)**	**m_{PZQ} (mg)**	**$n_{coformer}$ (mmol)**	**$m_{coformer}$ (mg)**
**PZQ·SAL·H_2O (1:1:1)**	0.557	174.1	0.558	77.1
**PZQ·3HBA (2:1)**	0.655	204.7	0.328	45.3
**PZQ·3HBA (1:1)**	0.554	173.1	0.553	76.4
**PZQ·4HBA (1:1)**	0.556	173.8	0.556	76.8
**PZQ·4ASA·MeCN (1:1:1)**	0.536	167.6	0.536	82.1
**PZQ·VAN (1:1)**	0.523	163.3	0.523	87.9
**PZQ·OXA (1:1)**	0.497	155.4	0.497	44.7
**PZQ·TRI·H_2O (1:2:2)**	0.341 [a]	106.6 [a]	0.682	143.4
**PZQ·BTC (2:1)**	0.568	177.5	0.284	72.2
**PZQ·5HIP·MeCN (1:4:2)**	0.240	75.0	0.960	175.0
**PZQ·SUB (2:1)**	0.640	200.0	0.320	56.3

a) n_{PZQ} and m_{PZQ} values for PZQ·0.5H_2O

Table S3. HPLC elution gradient

**Time (min)**	**Mobile phase A (%)** H_2O + 0.1% formic acid	**Mobile phase B (%)** MeCN + 0.1% formic acid
0.0	70	30
6	5	95
7	5	95
7.1	70	30
9.5	70	30

2. Powder X-ray diffraction

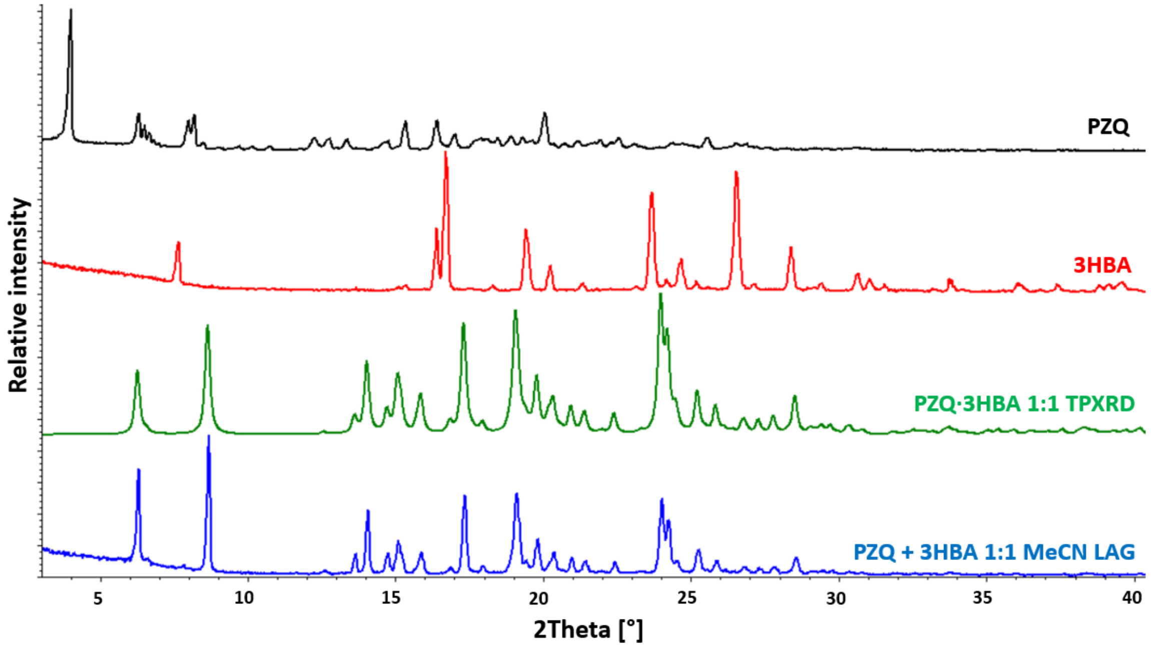

Figure S1. The comparison of powder diffractograms – praziquantel (PZQ, black line), 3-hydroxybenzoic acid (3HBA, red line), the theoretical powder pattern for **PZQ·3HBA 1:1** (green line) and powder pattern of material obtained by grinding PZQ and 3HBA in a 1:1 stoichiometric ratio with the addition of acetonitrile (blue line).

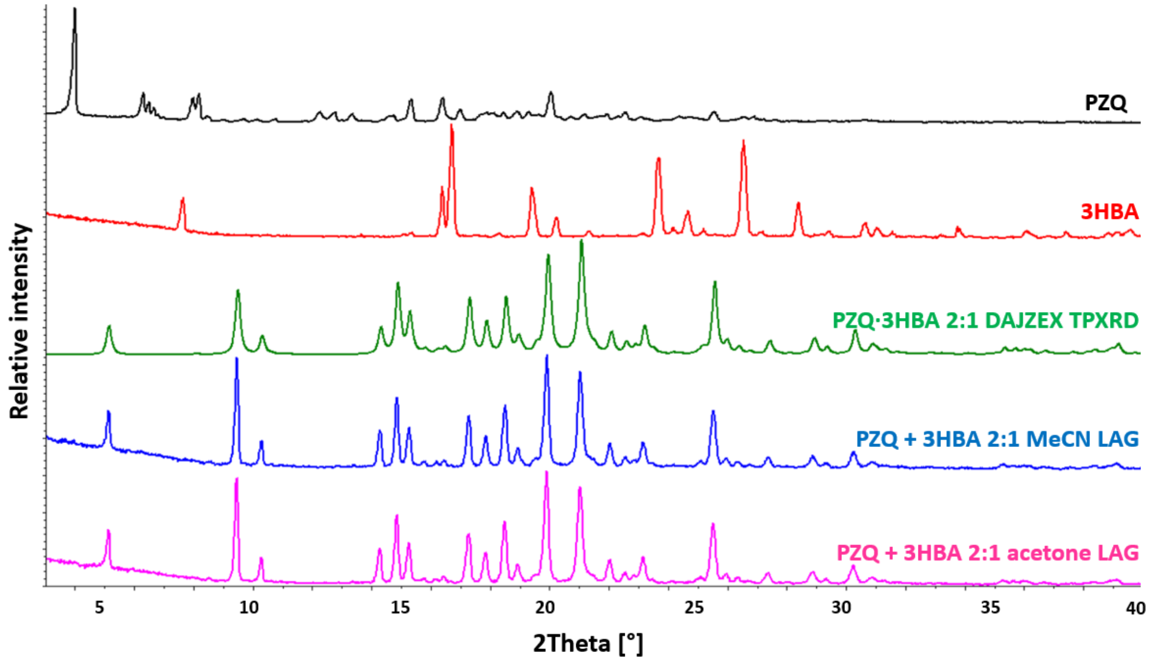

Figure S2. The comparison of powder diffractograms – praziquantel (PZQ, black line), 3-hydroxybenzoic acid (3HBA, red line), the theoretical powder pattern for **PZQ·3HBA 2:1** (green line) and powder patterns of materials obtained by grinding PZQ and 3HBA in a 2:1 stoichiometric ratio with the addition of acetonitrile (blue line) or acetone (pink line).

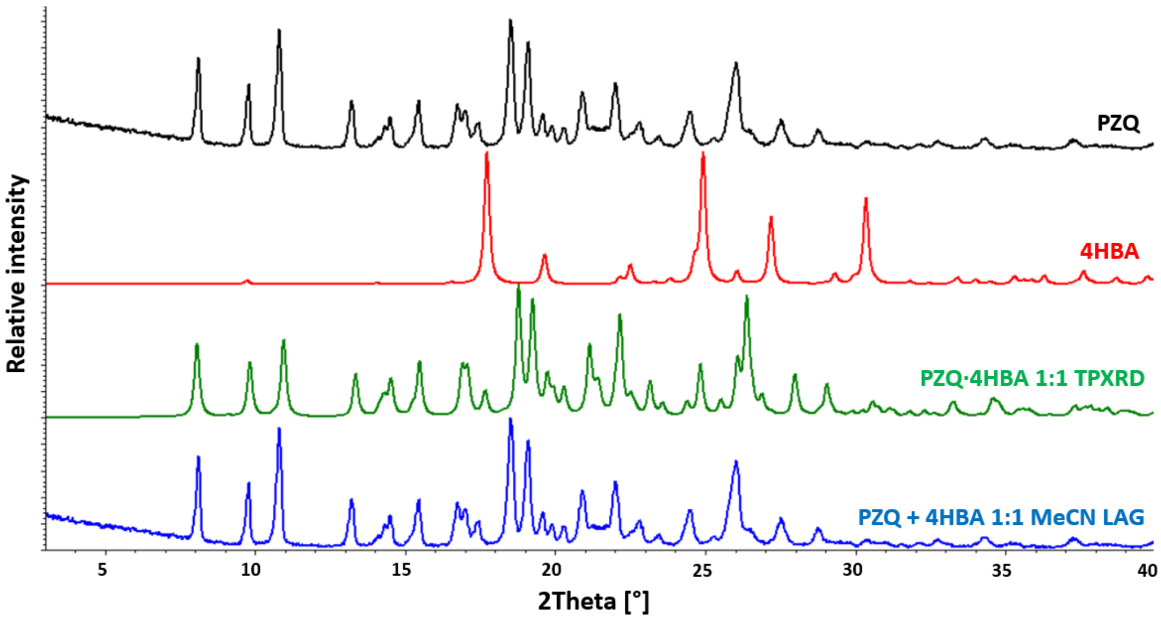

Figure S3. The comparison of powder diffractograms – praziquantel (PZQ, black line), 4-hydroxybenzoic acid (4HBA, red line), the theoretical powder pattern for **PZQ·4HBA 1:1** (green line) and powder pattern of material obtained by grinding PZQ and 4HBA in a 1:1 stoichiometric ratio with the addition of acetonitrile (blue line).

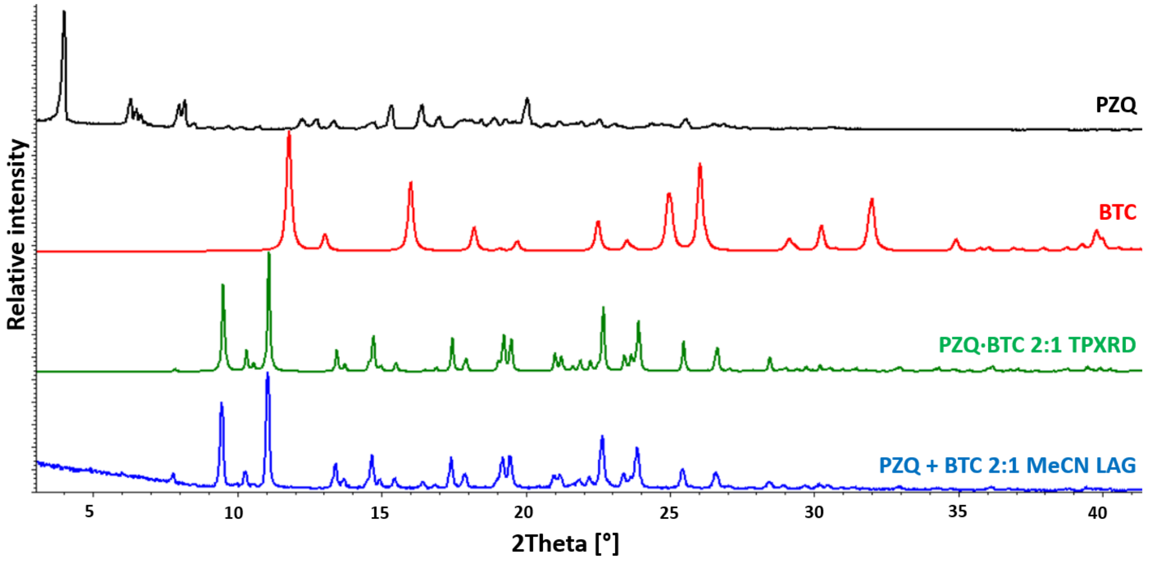

Figure S4. The comparison of powder diffractograms – praziquantel (PZQ, black line), benzene-1,2,4,5-tetracarboxylic acid (BTC, red line), the theoretical powder pattern for **PZQ·BTC** 2:1 (green line) and powder pattern of material obtained by grinding **PZQ** and **BTC** in a 2:1 stoichiometric ratio with the addition of acetonitrile (blue line).

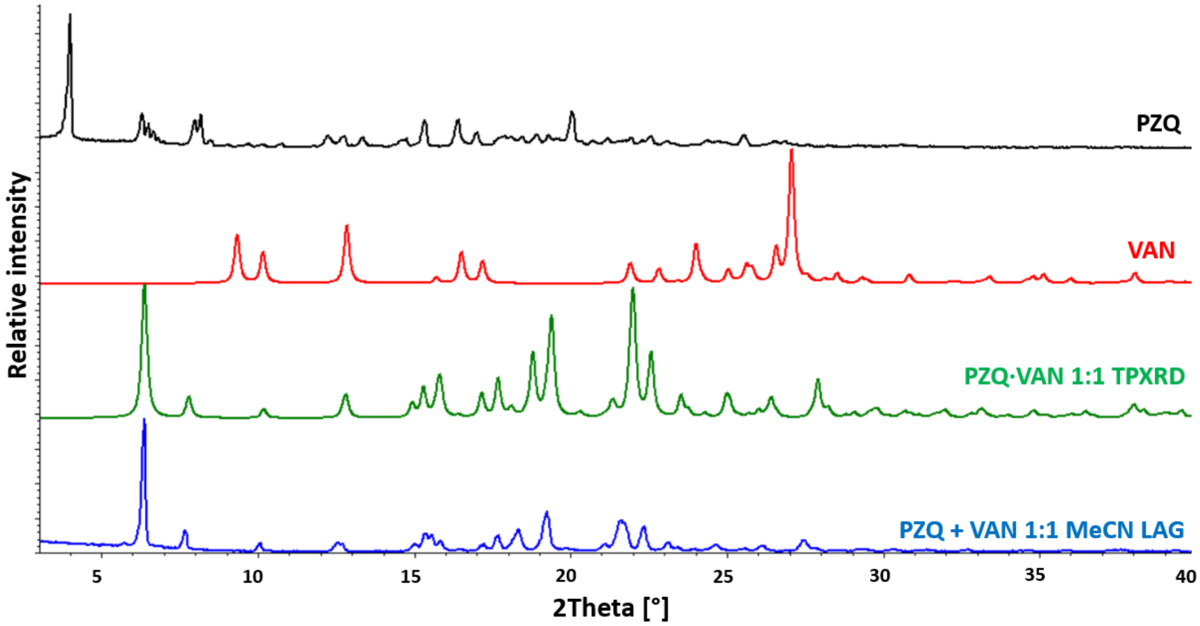

Figure S5. The comparison of powder diffractograms – praziquantel (PZQ, black line), vanillic acid (VAN, red line), the theoretical powder pattern for **PZQ·VAN 1:1** (green line) and powder pattern of material obtained by grinding PZQ and VAN in a 1:1 stoichiometric ratio with the addition of acetonitrile (blue line).

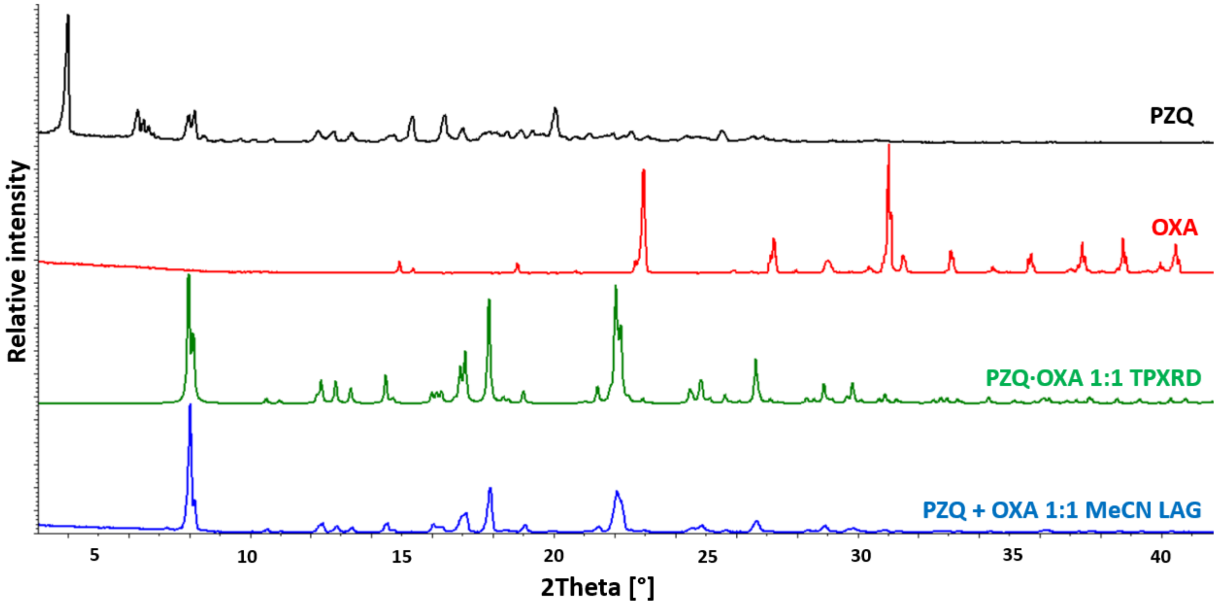

Figure S6. The comparison of powder diffractograms – praziquantel (PZQ, black line), oxalic acid (OXA, red line), the theoretical powder pattern for **PZQ·OXA 1:1** (green line) and powder pattern of material obtained by grinding PZQ and OXA in a 1:1 stoichiometric ratio with the addition of acetonitrile (blue line).

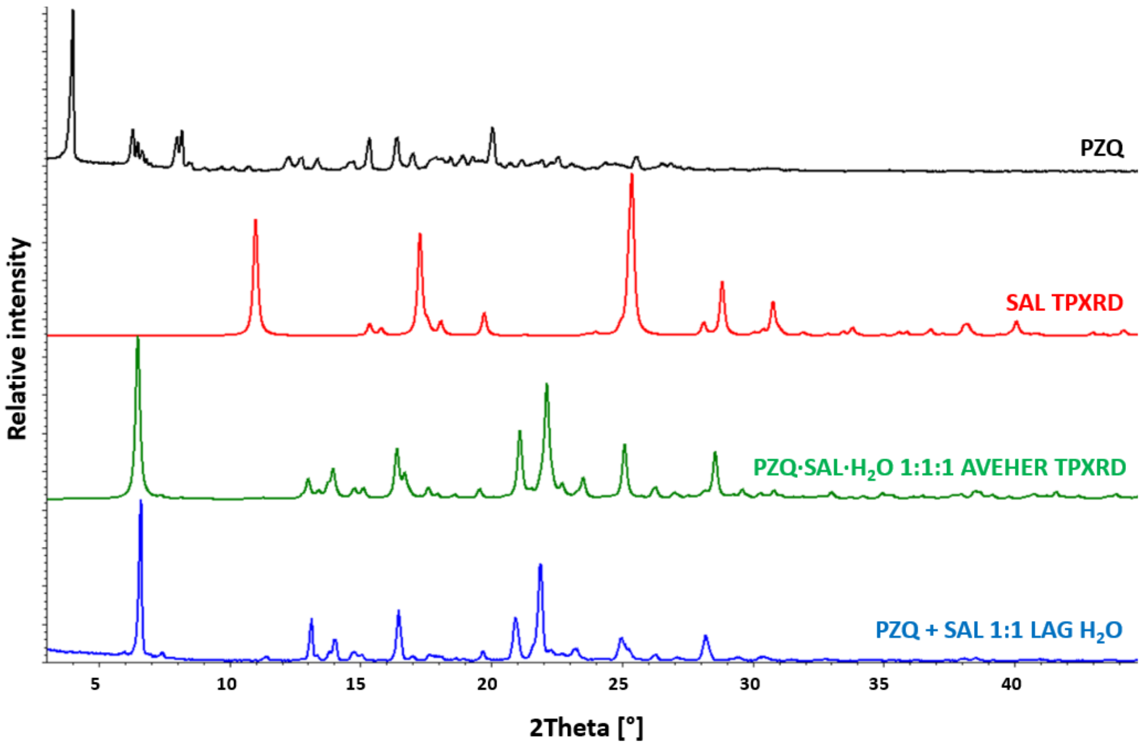

Figure S7. The comparison of powder diffractograms – praziquantel (PZQ, black line), salicylic acid (SAL, red line), the theoretical powder pattern for **PZQ·SAL·H_2O 1:1:1** (green line) and powder pattern of material obtained by grinding PZQ and SAL in a 1:1 stoichiometric ratio with the addition of acetonitrile (blue line).

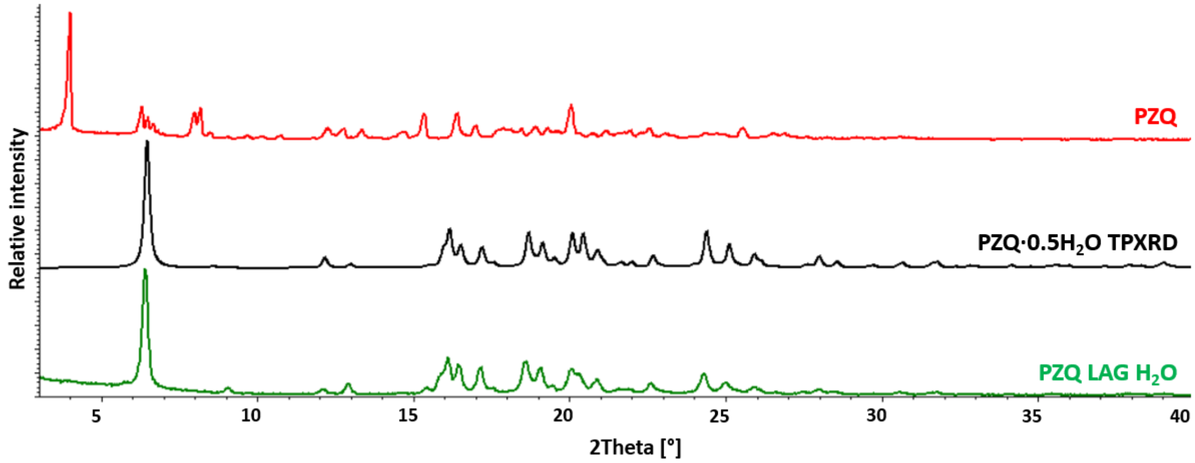

Figure S8. The comparison of powder diffractograms – praziquantel (PZQ, black line), the theoretical powder pattern for PZQ·0.5H_2O (black line) and powder pattern of material obtained by grinding PZQ with the addition of water (green line).

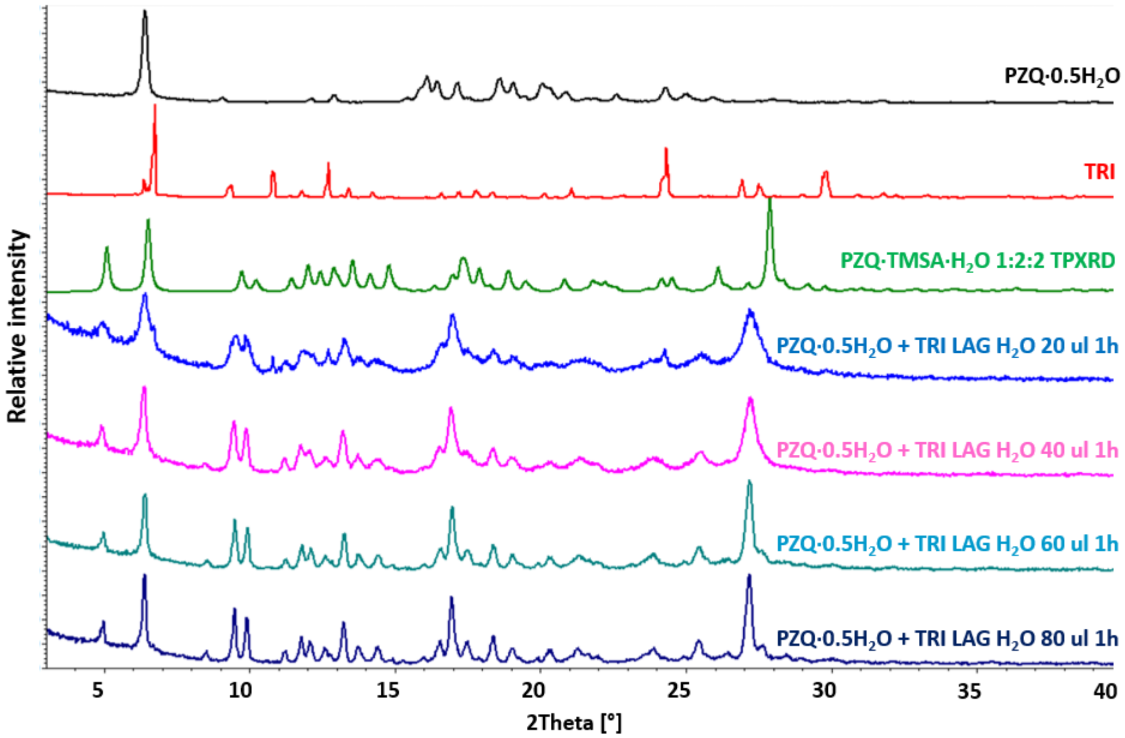

Figure S9. The comparison of powder diffractograms – praziquantel hemihydrate (PZQ·0.5H_2O, black line), trimesic acid (TRI, red line), the theoretical powder pattern for **PZQ·TRI·H_2O 1:2:2** (green line) and powder patterns of materials obtained by grinding PZQ·0.5H_2O and TRI in a 1:2 stoichiometric ratio with the addition of different volumes of water.

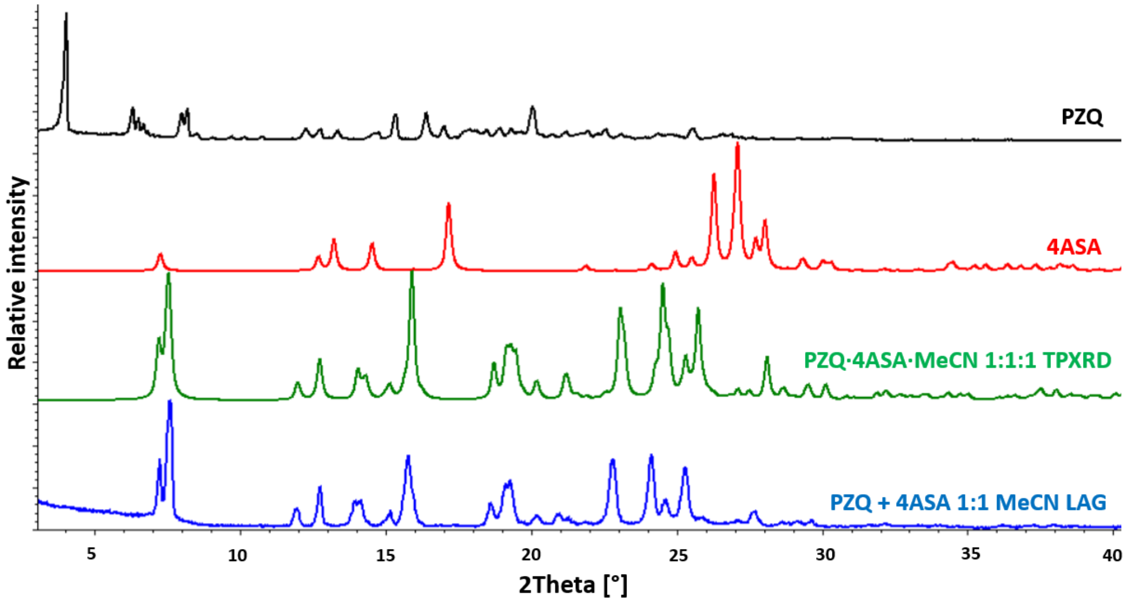

Figure S10. The comparison of powder diffractograms – praziquantel (PZQ, black line), 4-aminosalicylic acid (4ASA, red line), the theoretical powder pattern for **PZQ·4ASA·MeCN 1:1:1** (green line) and powder pattern of material obtained by grinding PZQ and 4ASA in a 1:1 stoichiometric ratio with the addition of acetonitrile (blue line).

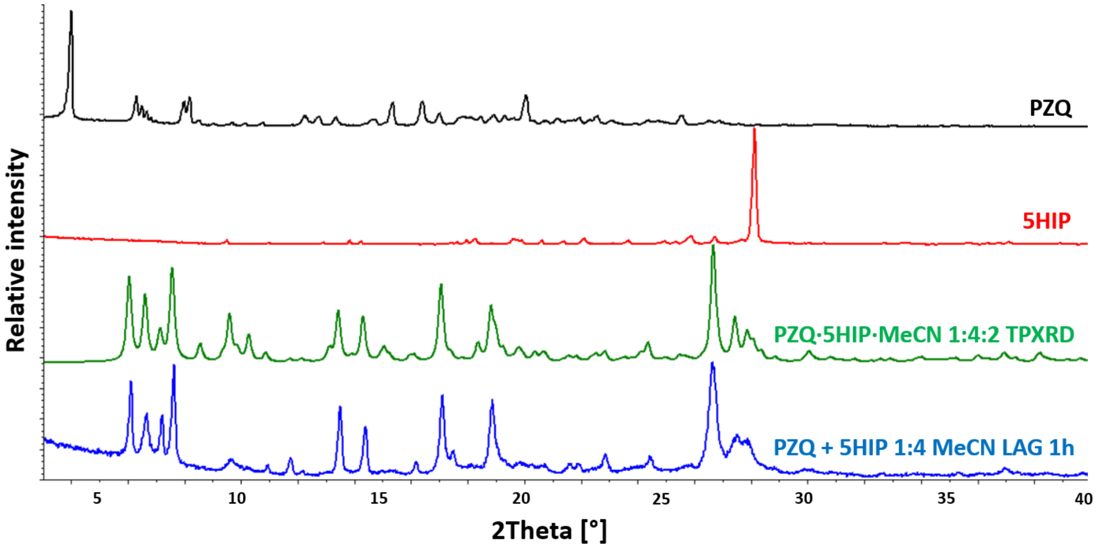

Figure S11. The comparison of powder diffractograms – praziquantel (PZQ, black line), 5-hydroxyisophthalic acid (5HIP, red line), the theoretical powder pattern for **PZQ·5HIP·MeCN 1:4:2** (green line) and powder pattern of material obtained by grinding PZQ and 5HIP in a 1:4 stoichiometric ratio with the addition of acetonitrile (blue line).

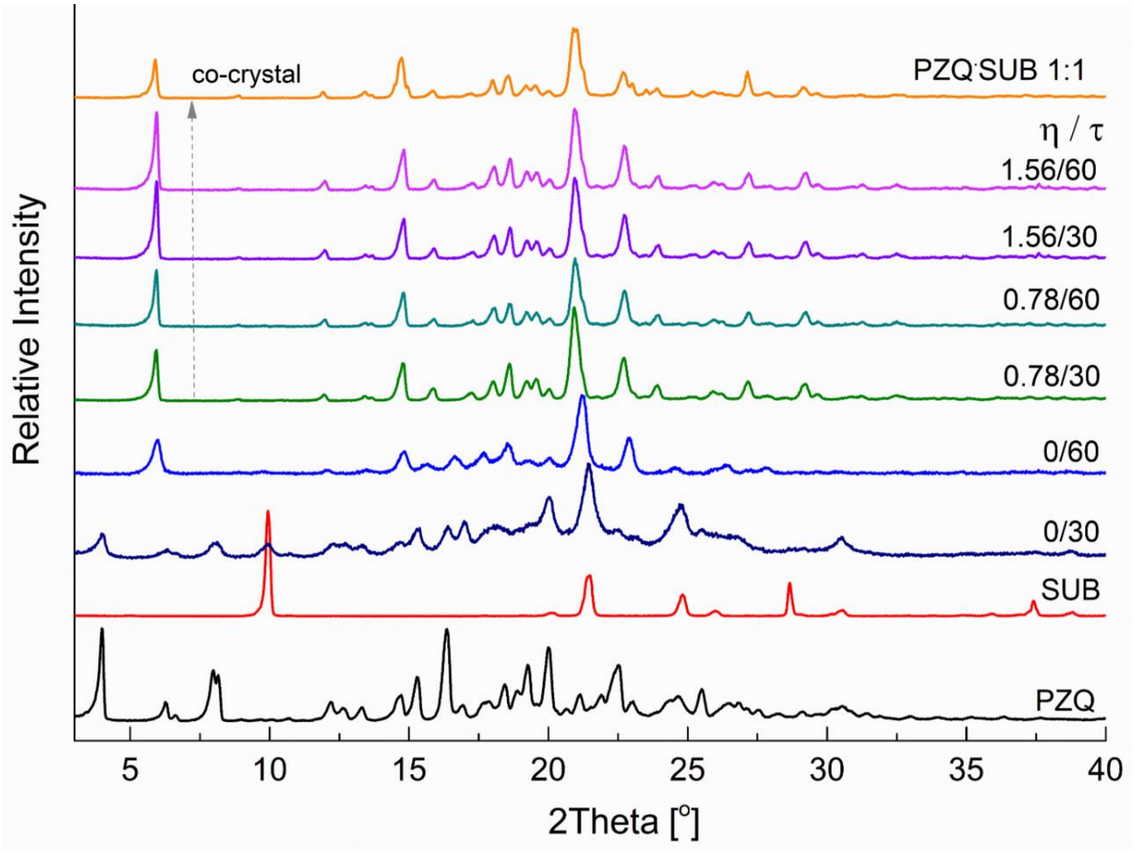

Figure S12. The comparison of powder diffractograms – praziquantel (PZQ, black line), suberic acid (SUB, red line), and powder patterns of the materials obtained by grinding PZQ and SUB in a 2:1 stoichiometric ratio at different solvent volume addition of ethanol/dichloromethane and 30-60 minutes time for grinding.

3. Crystallographic details

Table S4. Crystallographic data for obtained PZQ co-crystals.

**Cocrystal**	**PZQ·3HBA 1:1**	**PZQ·BTC 2:1**	**PZQ·SUB 2:1**
**Empirical formula**	$C_{26}H_{30}N_2O_5$	$C_{48}H_{54}N_4O_{12}$	$C_{23}H_{31}N_2O_4$
**Formula weight**	450.52	878.95	399.50
**Temperature/K**	293(2)	293(2)	293(2)
**Crystal system**	monoclinic	triclinic	monoclinic
**Space group**	$P2_1/n$	$P\bar{1}$	$I2/a$
**a/Å**	18.647(2)	9.5037(11)	26.0792(8)
**b/Å**	6.1392(7)	10.5666(13)	5.62250(10)
**c/Å**	20.483(2)	12.6738(13)	29.2260(8)
**α/°**	90	108.196(4)	90
**β/°**	92.694(10)	103.340(4)	90.836(3)
**γ/°**	90	106.032(3)	90
**V/Å^3**	2342.1(5)	1089.8(2)	4284.96(19)
**Z, Z'**	4, 1	1, 0.5	8
**ρ_{calc}/gcm^{-3}**	1.278	1.339	1.234
**μ/mm^{-1}**	0.089	0.097	0.084
**F(000)**	960	466	1709.0
**Crystal size/mm^3**	0.07 × 0.16 × 0.40	0.18 × 0.1 × 0.08	0.15 × 0.22 × 0.05
**Radiation/Å**	Mo Kα (λ = 0.71073)	Mo Kα (λ = 0.71073)	MoKα (λ = 0.71073)
**2θ range/°**	4.374 to 50.054	4.38 to 50.05	6.25 to 58.154
**Index ranges**	$-22 \leq h \leq 22$<br>$-7 \leq k \leq 7$<br>$-24 \leq l \leq 24$	$-11 \leq h \leq 11$<br>$-12 \leq k \leq 12$<br>$-15 \leq l \leq 15$	$-35 \leq h \leq 34$<br>$-7 \leq k \leq 7$<br>$-38 \leq l \leq 22$
**Reflections collected**	54450	43672	15527
**Independent reflections**	4128 [R_{int} = 0.0697, R_{sigma} = 0.0249]	3858 [R_{int} = 0.1061, R_{sigma} = 0.0459]	5048 [R_{int} = 0.0210, R_{sigma} = 0.0221]
**Data/restraints/parameters**	4128/0/301	3858/16/349	5048/0/276
**Final R indexes [I ≥ 2σ (I)]**	R_1 = 0.0693, wR_2 = 0.1232	R_1 = 0.0664, wR_2 = 0.1479	R_1 = 0.0598, wR_2 = 0.1798
**Final R indexes (all data)**	R_1 = 0.0791, wR_2 = 0.1275	R_1 = 0.0968, wR_2 = 0.1663	R_1 = 0.0816, wR_2 = 0.2054
**Goodness-of-fit on F^2**	1.26	1.03	0.93
**Largest diff. peak/hole/eÅ^{-3}**	0.13/-0.13	0.29/-0.41	0.42/-0.24
**CCDC deposit no.**	2314859	2314860	2252851

Table S5. Crystallographic data for obtained PZQ co-crystal solvates

Cocrystal solvate	PZQ·5HIP·MeCN 1:4:2	PZQ·TRI·H_2O 1:2:2
Empirical formula	$C_{55}H_{54}N_4O_{22}$	$C_{37}H_{40}N_2O_{16}$
Formula weight	1123.02	768.71
Temperature/K	293(2)	293(2)
Crystal system	Triclinic	monoclinic
Space group	$P\bar{1}$	$C2/c$
a/Å	13.2164(6)	16.49(4)
b/Å	13.4163(6)	34.59(8)
c/Å	15.6478(7)	14.00(4)
α/°	85.148(4)	90
β/°	69.527(4)	116.82(6)
γ/°	89.855(4)	90
V/Å^3	2588.9(2)	7128(30)
Z, Z'	2, 1	8, 1
ρ_{calc}/gcm^{-3}	1.441	1.433
μ/mm^{-1}	0.113	0.113
F(000)	1176	3232
Crystal size/mm^3	0.18 × 0.10 × 0.06	0.16 × 0.05 × 0.04
Radiation/Å	Mo Kα (λ = 0.71073)	Mo Kα (λ = 0.71073)
2θ range/°	3.944 to 49.948	4.022 to 36.992
Index ranges	$-15 \le h \le 15$<br>$-15 \le k \le 15$<br>$-18 \le l \le 18$	$-14 \le h \le 14$<br>$-30 \le k \le 30$<br>$-12 \le l \le 12$
Reflections collected	145389	23020
Independent reflections	9096<br>[R_{int} = 0.1564, R_{sigma} = 0.0549]	2664<br>[R_{int} = 0.0995, R_{sigma} = 0.0442]
Data/restraints/parameters	9096/0/694	2664/8/522
Final R indexes [I ≥ 2σ (I)]	R_1 = 0.1574, wR_2 = 0.2830	R_1 = 0.0810, wR_2 = 0.2186
Final R indexes (all data)	R_1 = 0.1750, wR_2 = 0.2923	R_1 = 0.1039, wR_2 = 0.2415
Goodness-of-fit on F^2	1.33	1.06
Largest diff. peak/hole/eÅ^{-3}	0.34/-0.35	0.37/-0.21
CCDC deposit no.	2314861	2314858

4. Hydrogen-bonding details

Table S6. Hydrogen bonds formed by PZQ and 3HBA in the **PZQ·3HBA** 1:1 cocrystal.

D—H···*A*	*D*—H (Å)	H···*A* (Å)	*D*···*A* (Å)	*D*—H···*A* (°)
O5—H5···O1	0.82	1.84	2.655(2)	171.2
O4—H4···O3^{i}	0.82	1.81	2.629(2)	174.5
Symmetry code: (i) −*x*, −*y*+4, −*z*+1.				

Table S7. Hydrogen bonds formed by PZQ and BTC in the **PZQ·BTC** 2:1 cocrystal.

D—H···*A*	*D*—H (Å)	H···*A* (Å)	*D*···*A* (Å)	*D*—H···*A* (°)
O3B—H3B···O1^{i}	0.82	1.84	2.620(5)	158.8
O4B—H4BA···O1^{i}	0.82(1)	1.83(1)	2.650(4)	174(8)
O5B—H5BA···O2	0.82(1)	1.89(2)	2.682(7)	161(7)
O6B—H6BA···O2^{i}	0.82(1)	1.93(2)	2.717(5)	162(5)
Symmetry code(s): (i) *x*, *y*−1, *z*−1. The O3B, O4B, O5B, O6B oxygen atoms and H3B, H4BA, H5BA, H6BA hydrogen atoms with fixed occupancies of 0.50.				

Table S8. Geometry and type of hydrogen bonds formed by PZQ and 5HIP molecules in the **PZQ·5HIP·MeCN 1:4:2**.

Type of interaction	*D*—H···*A*	*D*—H (Å)	H···*A* (Å)	*D*···*A* (Å)	*D*—H···*A* (°)
Carboxylic homosynthons $R_2^2(8)$ formed by O-$H_{carboxyl}$···O=$C_{carboxyl}$	O4A—H4AA···O3B^{ii}	0.82	1.84	2.650(6)	171.8
	O4B—H4BA···O3A^{iii}	0.82	1.83	2.642(6)	167.8
	O4C—H4C···O3D^{ii}	0.82	1.82	2.626(6)	168.2
	O4D—H4D···O3C^{iii}	0.82	1.82	2.633(6)	173.5
	O6A—H6AA···O5B^{i}	0.80(8)	1.85(8)	2.640(6)	172(8)
	O6B—H6BA···O5A^{iv}	0.78(9)	1.86(9)	2.638(6)	175(10)
	O6C—H6C···O5D^{i}	0.82	1.86	2.671(6)	172.5
	O6D—H6D···O5C^{iv}	0.82	1.81	2.630(6)	174.0
O—$H_{hydroxyl}$···O=C_{PZQ}	O7A—H7A···O1	0.82	1.75	2.564(6)	171.2
	O7C—H7C···O2	0.82	1.88	2.641(7)	154.9
O—$H_{hydroxyl}$···O—$H_{hydroxyl}$	O7B—H7B···O7A	0.82	1.89	2.694(6)	167.9
	O7D—H7D···O7C	0.82	1.94	2.711(6)	157.5
Symmetry code(s): (i) *x*+1, *y*, *z*; (ii) *x*, *y*, *z*+1; (iii) *x*, *y*, *z*−1; (iv) *x*−1, *y*, *z*.					

Table S9. Hydrogen bonds in the **PZQ·TRI·H_2O 1:2:2**.

D—H···*A*	*D*—H (Å)	H···*A* (Å)	*D*···*A* (Å)	*D*—H···*A* (°)
O8B—H8B···O5B^{i}	0.82	1.80	2.606(9)	167.6
O8A—H8A···O5A^{ii}	0.82	1.77	2.574(8)	165.2
O6A—H6AA···O7A^{iii}	0.82	1.77	2.546(8)	155.9
O6B—H6BA···O7B^{iv}	0.82	1.75	2.553(9)	165.9
O4A—H4AA···O1	0.82	1.74	2.545(10)	166.3
O4B—H4BA···O10A	0.82	1.72	2.490(15)	156.6
O10A—H10A···O9	0.85	1.97	2.767(17)	154.9
O9—H9A···O1	0.97	2.11	3.039(14)	161.2
O9—H9B···O3B	0.82	2.10	2.860(14)	154.3
O10B—H10C···O10A	0.85	1.90	2.71(2)	159.1
O10B—H10D···O2^{v}	0.85	2.03	2.73(3)	138.5
Symmetry code(s): (i) x+1/2, −y+1/2, z+1/2; (ii) x−1/2, −y+3/2, z−1/2; (iii) x+1/2, −y+3/2, z+1/2; (iv) x−1/2, −y+1/2, z−1/2; (v) x+1, y, z+1.				

Table S10. Hydrogen bonds formed by PZQ with SUB in the **PZQ·SUB** 2:1 co-crystal.

D—H···*A*	*D*—H (Å)	H···*A* (Å)	*D*···*A* (Å)	*D*—H···*A* (°)
O3—H3···O1	0.82	1.817(2)	2.622(3)	166.8(2)
C11—H11B···O4	0.97	2.522(1)	3.406(1)	151.5(1)
C11—H11A···O2	0.97	2.530(2)	3.427(2)	153.6(1)
C9—H9···O1	0.98	2.412(3)	3.382(4)	170.5(3)
C1—H1A···O1	0.97	2.683(2)	3.578(1)	153.7(3)
O3—H3···C12	0.82	2.765(1)	3.574(2)	169.3(1)
C14—H14A···C4 (C-H···π)	0.97	2.825(4)	3.784(1)	170.1(2)

5. Physical stability of PZQ·SUB 2:1 co-crystal and of PZQ·SUB physical mixture.

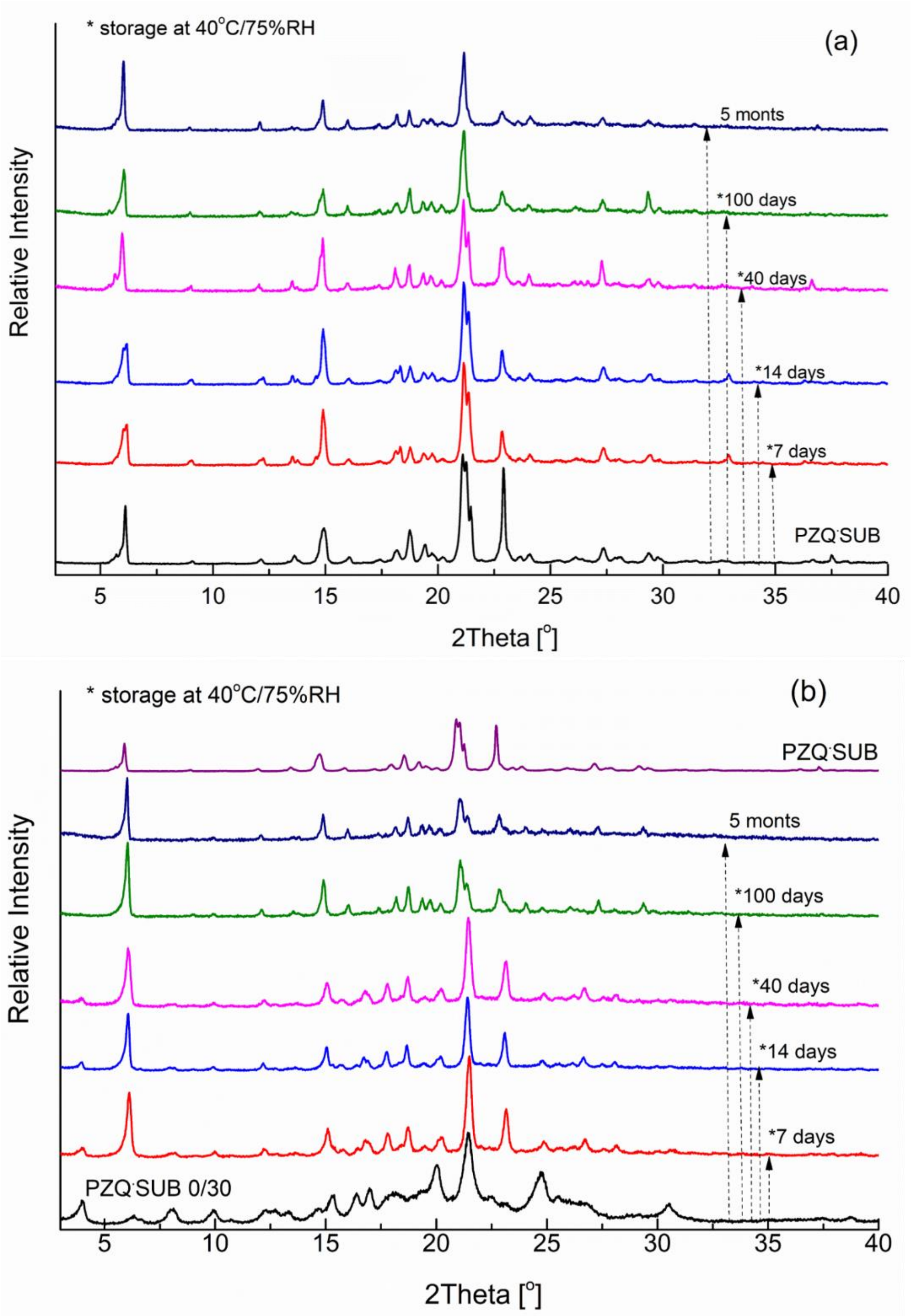

Figure S13. XRD patterns of (a) co-crystal PZQ·SUB, and (b) of the mixture of PZQ-SUB, after several periods of storage time in extreme climatic conditions.

6. XPS details for PZQ·SUB 2:1 co-crystal

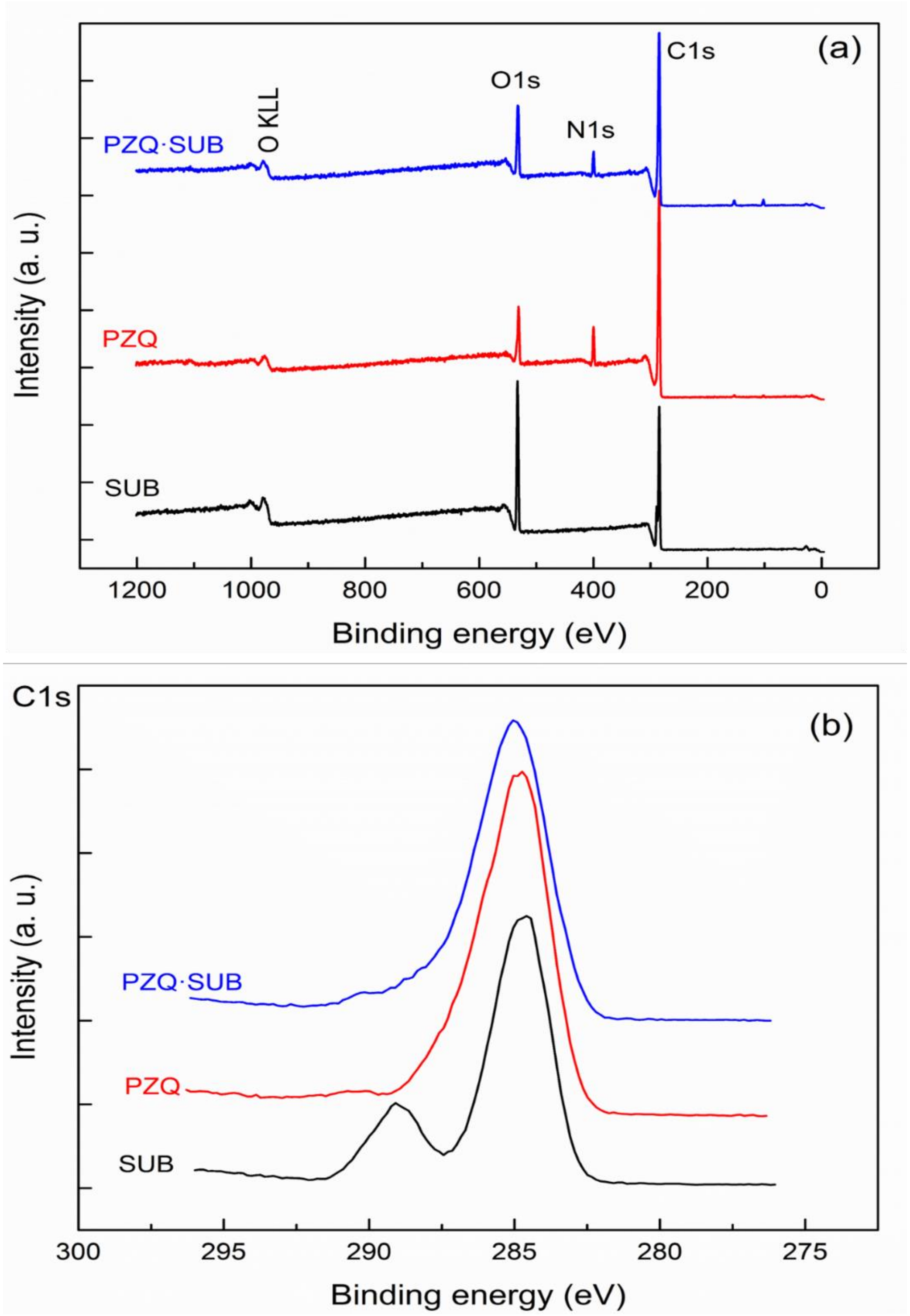

Fig. S14. (a) XPS survey spectra and (b) C1s high resolution spectra, of **PZQ**, **SUB**, and **PZQ·SUB**.

7. Solubility details for PZQ co-crystals.

Table S11. The concentration of PZQ in micrograms per milliliter ([PZQ] μg/mL) in each dissolution medium for each co-crystal, attained in 24 hours.

Co-Crystal No.	Compound Name / Dissolution medium	H_2O [PZQ] (μg/mL)	SGF [PZQ] (μg/mL)	SIF [PZQ] (μg/mL)	SCF [PZQ] (μg/mL)
**1**	PZQ – 5-HYDROXYISOPHTHALIC ACID (5HIP) – MeCN	95.51	85.70	361.00	122.86
**2**	PZQ – VANILLIC ACID (VAN)	266.00	246.37	281.11	362.05
**3**	PZQ – 4-AMINOSALICYLIC ACID (4ASA) – MeCN	278.89	323.31	264.29	294.31
**4**	PZQ – TRIMESIC ACID (TRI) – H_2O	56.09	44.98	242.32	193.95
**5**	PZQ – 4-HYDROXYBENZOIC ACID (4HBA)	225.06	203.21	217.86	247.22
**6**	PZQ – SALICYLIC ACID (SAL) – H_2O	220.38	202.80	246.81	258.43
**7**	PZQ – 3-HYDROXYBENZOIC ACID (3HBA) (1:1)	252.60	214.21	232.31	251.67
**8**	PZQ – OXALIC ACID (OXA)	250.12	238.78	229.26	252.98
**9**	PZQ – 3-HYDROXYBENZOIC ACID (3HBA) (2:1)	248.12	231.45	226.47	257.95
**10**	PZQ – BENZENE-1,2,4,5-TETRACARBOXYLIC ACID (BTC)	354.90	217.19	317.60	260.75
**11**	PZQ – SUBERIC ACID (SUB)	271.99	278.27	226.99	250.99
**12**	PZQ	210.96	200.30	193.04	220.01

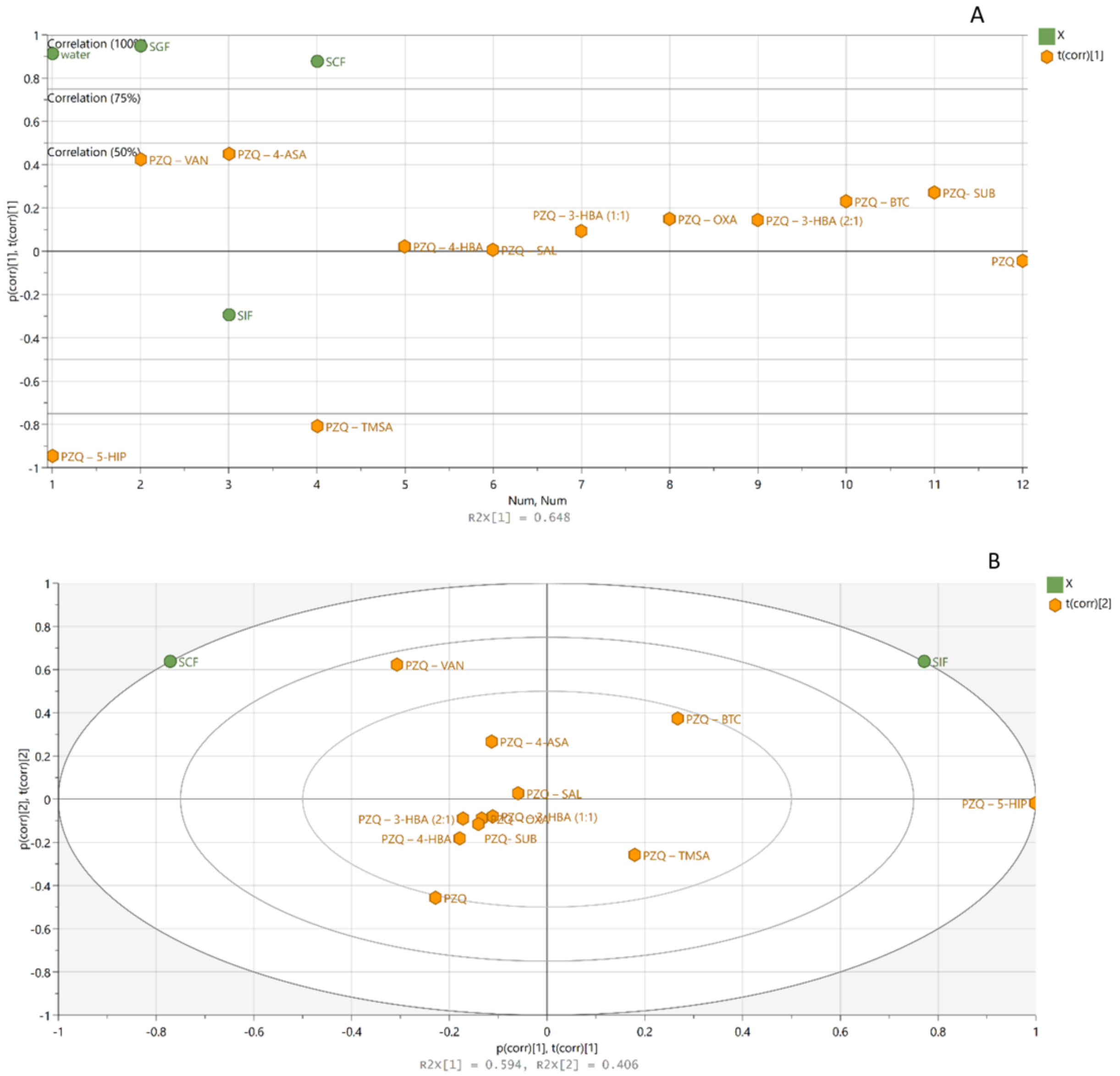

Figure S15.. Biplots of PCA models on equilibrium solubility data of studied co-crystals considering all (A.) or only SIF and SCF (B.) as biorelevant dissolution media.
